# Reference ranges for clinical electrophysiology of vision

**DOI:** 10.1007/s10633-021-09831-1

**Published:** 2021-04-21

**Authors:** C. Quentin Davis, Ruth Hamilton

**Affiliations:** 1 LKC Technologies, Gaithersburg, MD USA; 2 Department of Clinical Physics and Bioengineering, Royal Hospital for Children, NHS Greater Glasgow and Clyde, Glasgow, UK; 3 College of Medical, Veterinary and Life Sciences, University of Glasgow, Glasgow, UK

**Keywords:** Reference data, Reference limit, Reference interval, Normative data, ISCEV standard, ERG, VEP, EOG

## Abstract

**Introduction:**

Establishing robust reference intervals for clinical procedures has received much attention from international clinical laboratories, with approved guidelines. Physiological measurement laboratories have given this topic less attention; however, most of the principles are transferable.

**Methods:**

Herein, we summarise those principles and expand them to cover bilateral measurements and one-tailed reference intervals, which are common issues for those interpreting clinical visual electrophysiology tests such as electroretinograms (ERGs), visual evoked potentials (VEPs) and electrooculograms (EOGs).

**Results:**

The gold standard process of establishing and defining reference intervals, which are adequately reliable, entails collecting data from a minimum of 120 suitable reference individuals for each partition (e.g. sex, age) and defining limits with nonparametric methods. Parametric techniques may be used under some conditions. A brief outline of methods for defining reference limits from patient data (indirect sampling) is given. Reference intervals established elsewhere, or with older protocols, can be transferred or verified with as few as 40 and 20 suitable reference individuals, respectively. Consideration is given to small numbers of reference subjects, interpretation of serial measurements using subject-based reference values, multidimensional reference regions and age-dependent reference values. Bilateral measurements, despite their correlation, can be used to improve reference intervals although additional care is required in computing the confidence in the reference interval or the reference interval itself when bilateral measurements are only available from some of subjects.

**Discussion:**

Good quality reference limits minimise false-positive and false-negative results, thereby maximising the clinical utility and patient benefit. Quality indicators include using appropriately sized reference datasets with appropriate numerical handling for reporting; using subject-based reference limits where appropriate; and limiting tests for each patient to only those which are clinically indicated, independent and highly discriminating.

**Supplementary Information:**

The online version contains supplementary material available at 10.1007/s10633-021-09831-1.

## Introduction

Reference^1^ values describe the diversity observed in parameters measured from a group of individuals representing some healthy population. Improved diagnostic quality results from using reference values garnered from an adequately sized sample of appropriate reference individuals. This process has been the subject of extensive international cooperative work in the fields of laboratory medicine [2–5], and human biometrics such as height and weight [6] have received some attention in other areas of clinical measurement [7, 8], but less so in clinical electrophysiology of vision.

The International Society for Clinical Electrophysiology of Vision (ISCEV) standards [9–13] and guidelines [14, 15] state the need for reference values, but it is not within the scope of such documents to provide detail on the process. Similarly, whilst some medical devices for visual electrophysiology hold in-built reference data, techniques for verifying their suitability for a patient population may not be included. The purpose of this work is to collate expertise from other clinical scientific areas as well as our own computational studies and present a guide to reference values relevant for those undertaking or interpreting clinical visual electrophysiology tests. This work is also pertinent to other clinical measurements on bilateral systems (e.g. hearing, nerve conduction) where intra-subject correlation needs to be considered.

Typically, a reference interval for a single parameter includes 95% of its reference values. This 95% figure may be based on the 5% significance level, widely used since the early twentieth century, and selected on the basis of convenience for judging the significance of a deviation [16]. More stringent criteria such as a 99.8% reference range have been proposed [17], but are not widely used nor included in any consensus guidelines. The use of a 95% reference range in reporting clinical test results means that any single test parameter has a 1 in 20 chance of being classified as abnormal when no abnormality exits. When multiple parameters per test (e.g. a- and b-wave amplitudes and peak times) are analysed, or when multiple tests (e.g. full-field ERG, pattern VEP, pattern ERG) are conducted, the chance of any false-positive finding rises, albeit related to the extent of independence of test parameters [18–20]. Reference limits can be adjusted to reduce this risk (see section *Adjusting for multiple measurements*); however, the correlation between the measures must be known. It is advisable to limit electrophysiology tests to only those clinically indicated—preferably both independent and highly discriminating—rather than conducting a standard battery of tests on every patient. This reduces false-positive findings [21], limits additional unnecessary testing, reduces patient risk from investigations or therapeutic interventions, reduces patient anxiety and reduces resource wastage in health care [20].

**Fig. 1:**
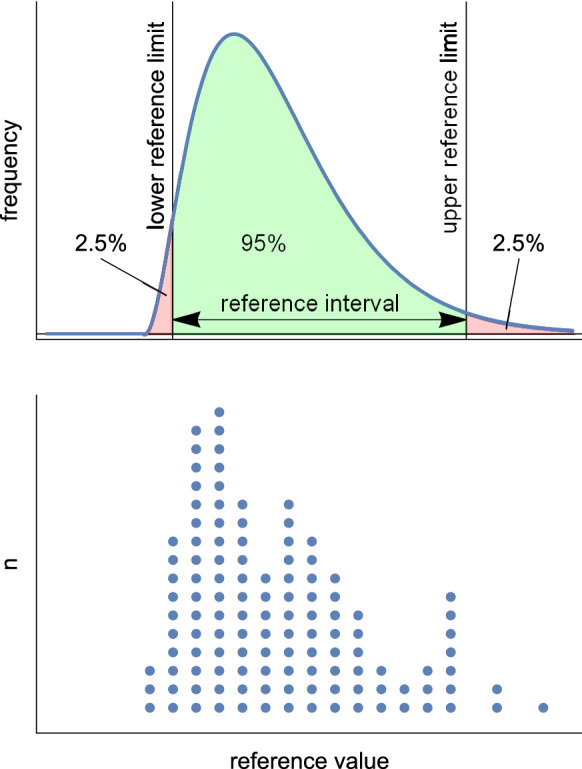
Illustration of terms. Upper panel: example of the distribution of reference values from the reference population shown as a probability density function (idealised data, demonstrated herein with a gamma distribution. The gamma distribution is one of many probability distributions in an exponential family; others include the normal (Gaussian), log-normal and Poisson distributions. It was selected to illustrate a skewed distribution as well as the issues associated with having a mismatch between a fitted model and the underlying data in parametric methods). The reference interval spans from the lower to the upper reference limit and encloses the middle 95% of the distribution. Lower panel: histogram of 120 random measurements sampled from the distribution in the upper panel, forming the reference distribution. The reference intervals and reference limits are derived from sample measurements such as these, along with estimates of the uncertainties of those limits

The following terminology has been established for the subject of reference values and is endorsed by the World Health Organization [2]:

- Reference individual—a subject who meets the inclusion criteria.
- Reference population—the group comprising all reference individuals who exist, usually an unknown quantity.
- Reference sample group—the group of reference individuals selected, usually non-randomly, to represent the reference population.
- Reference value—value of a test parameter measured from a reference individual (Fig. 1).
- Reference distribution—the frequency of all reference values (Fig. 1). Often this distribution cannot be described by a single mathematical function. It is relatively rare to find a Gaussian distribution, so defining reference limits as the mean ± some standard deviations of the reference values is rarely appropriate and risks systematic misclassifications (see *Parametric method*).
- Reference limit—a value derived mathematically from the reference distribution, defined such that a stated fraction (e.g. 2.5%) of the reference values lies above or below it (Fig. 1).
- Reference interval—the interval considered as healthy, which for two-tailed limits is the interval between and including the two reference limits (Fig. 1) or for one-tailed reference limits, the values equal to or above/below the one reference limit.

Commercial software and freeware [22] are available to undertake most or all of the processes described here. Our computational studies, including all figures, were performed in Mathematica® version 12.2 (Wolfram Research, Champaign, IL, USA); a copy of the source code is available as supplementary material.

## Establishing reference intervals: direct sampling

### Defining reference individuals

Direct sampling refers to reference individuals selected from a reference population using specific, well-defined criteria. The reference population is defined using criteria such that it is similar to the patient group in aspects such as age, ethnicity and gender: a single group of young, healthy adults is unlikely to be as clinically appropriate as age-related reference intervals [2]. Group comparisons in a research context should also ensure balanced ages, ethnicities and genders between disease groups and control or comparison groups. Careful selection of the reference population is important: a too-narrowly defined population with many restrictions will have only limited applicability. Including even a few diseased subjects in the reference sample group, either by a definitional oversight or misdiagnosis, may have a marked effect on the reference interval. For example, suppose a disease decreases an ERG measurement to abnormal levels. If there were 100 subjects in the reference sample group, the 2.5th percentile would be the value from the subject with the 3rd smallest result when using the nonparametric method (see footnote 2: index = 0.5 + *p n* = 0.5 + (0.025 × 100) = 3). If three subjects had the disease, the reference limit would be set by one of the disease cases, not someone free from disease. With two diseased subjects, the reference limit would be the value from the free-from-disease subject with the smallest result, rather than from the free-from-disease subject with the 3rd smallest result.

For a priori and a posteriori population sampling, criteria are applied before and after data collection, respectively. Exclusion criteria are used to minimise the number of subjects with non-pathology-related changes and may differ by test and centre (Table 1). A further list of factors known to affect ISCEV standard parameters, i.e. potential partitioning criteria, is also given in Table 1.

**Table 1:** Exclusion criteria and partitioning factors for consideration when designing a reference data study for clinical visual electrophysiology

Exclusion criteria for consideration when selecting reference individuals	Potential partitioning factors for consideration
Restricted diet	Age
Alcohol use	Sex
Drug use	Affluence/deprivation
Drug misuse	Ethnicity/pigmentation
Recent or current illness	Refractive status
History of premature birth	
History or family history of ophthalmic or neurological disease	
History of retinal surgeries, recent other ocular surgeries (e.g. cataract)	
Indicators of ocular disease such as high intraocular pressure, diabetes, poor cup-to-disc ratios, poor best-corrected visual acuity	

These exclusion criteria define subjects eligible for recruitment to a reference study. For any study, ethical approval and relevant permissions are required, and subjects must give written, informed consent. Monitoring data findings and adjusting recruitment strategies ensure adequate demographic and age distributions. Partition factors and exclusion criteria are included in a questionnaire with any additional relevant factors to capture a minimum dataset for recruited subjects. Specific questions relating to the presence of or family history of ophthalmic or neurological conditions are valuable: self-reporting, screening or ophthalmic examination may be warranted, and assessment is adapted to be suitable for age. Further details captured at the time of testing include test site, time of day, order of tests, person performing the test, equipment serial numbers, protocol identification numbers, stimulus calibration information, device and electrode type. Any factors that deviate from the relevant ISCEV standard is noted, and where the standard allows options (for example, ERG active electrode type) or a range of variables, the option or value chosen is noted.

### Nonparametric method

The nonparametric method is the gold standard for establishing reference limits [2]. It makes no assumptions about the shape of the reference distribution, relying on only the values near the edges of the frequency plot (Fig. 1). Data are ranked and percentiles calculated,^2^ with the minimum number of data points, *n*, required to distinguish two adjacent percentiles separated by *P*% given by

1 $$n = \left( \frac{100}{P} \right) - 1$$

Therefore, to distinguish the 2.5th from the 5th percentile, a minimum of *n* = 39 data points are required. With this minimum number and without using interpolation techniques, the extreme values of the distribution become the estimated reference limits and are therefore vulnerable to aberrant values. Increasing the sample size reduces this vulnerability.

Precision of a reference limit is conventionally expressed as its 90% confidence interval (CI) and can be calculated from ranked data [25] or bootstrapping (see below). A sample size of 120 data points is the smallest number that allows exact, nonparametric calculation of the precision of each reference limit [26] and therefore is the minimum recommended [2]. This sample size is after any outlier removal and is for each partition (e.g. for males and for females if these differ significantly).

### Parametric method

Unlike nonparametric methods, parametric methods use information from all the reference values, thereby reducing uncertainty but relying on assumptions about the shape of the underlying reference population. Physiological measurement data seldom have a Gaussian distribution, if for no other reason than Gaussians have nonzero probability for all values, which would include physiologically impossible negative time delays (where the response occurs before the stimulus) as well as mathematically impossible negative peak-to-peak amplitudes. The central limit theorem, which makes many statistical processes which are sums have a Gaussian distribution, applies only to the centre and not to the tails of a distribution which is where the reference limits are located.

**Fig. 2:**
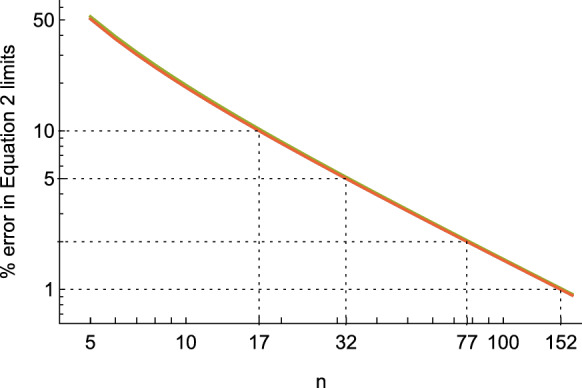
Percentage error in Eq. 2 relative to the non-central Student’s T distribution of the Lawless interval, as a function of number of data points* n*. Equation 2 provides the confidence intervals for the upper and lower reference limits. All four confidence interval points are shown, although results overlap. Error between Eq. 2 and the non-central Student’s T distribution falls as 1/n

Standard tests such as Kolmogorov–Smirnov or Anderson–Darling’s [27] can be applied to assess normality. If acceptably normal, 95% reference limits are defined as the sample mean ± 1.96 standard deviations. The 90% CI of each reference limit can be calculated as

2 $$90\% {\text{CI}} = {\text{reference limit}} \pm 2.81\left( {\frac{s}{\sqrt n }} \right)$$

where *s* is the standard deviation of the sample and *n* is the number of data points [25]. This formula is an approximation to the non-central Student’s T distribution of the Lawless interval [28]. The error between the formulae is shown in Fig. 2, where for reasonably sized *n*, the difference is sufficiently small that they are interchangeable (and Eq. 2 is much easier to calculate). Because parametric tests involve more assumptions than nonparametric tests, they are generally more powerful and require smaller sample sizes to reach equivalent certainty as the nonparametric gold standard [29].

**Fig. 3:**
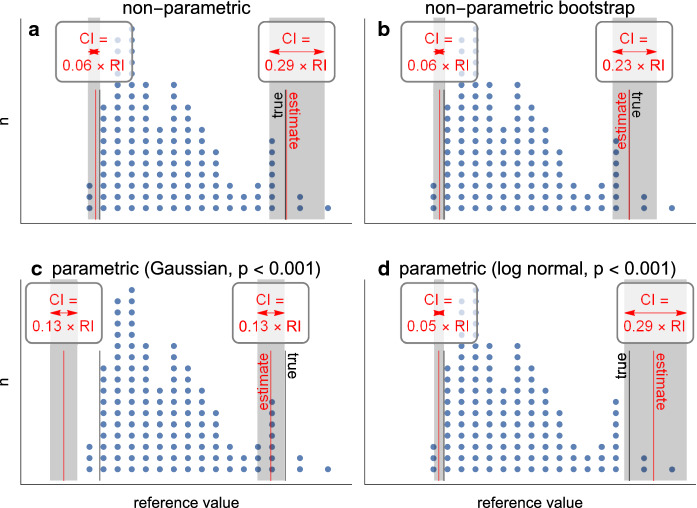
Illustration of nonparametric **(a, b)** and parametric **(c, d)** reference interval estimates with precision estimates (90% CI). Data are from Fig. 1. Black vertical lines: true reference limits of the underlying population calculated exactly by integrating the probability density function of the continuous gamma distribution used as the source for the sampled data. Dots: sampled data from underlying population. Red vertical lines: reference limits estimated from sample data using the different methods. Grey boxes: 90% CIs of estimated reference limits. For nonparametric estimates **(a, b)**, CIs are wider for the longer (right) tail of the distribution, being 29% of the reference interval in panel **a**, exceeding the 20% goal so that more measurements may be needed. Bootstrapping (1000 × , panel **b** narrows this CI from 29 to 23% of the reference interval. Estimated limits are close to true limits. Panel **c** shows parametric (mean ± 1.96 standard deviations) estimates and their CIs. The data do not have a Gaussian distribution (normality test fail, *p* < 0.05). 90% CIs are incorrectly symmetrical for both reference limits, and inaccurately narrow (13% of the reference interval): the lower CI does not enclose the true reference limit. Panel **d** shows parametric estimates, performed on log-transformed data, and back-transformed for display. Estimated limits’ 90% CIs enclose the true limits, but the precision of longer (right) tail is 29% of the reference interval, exceeding the 20% goal so that more measurements may be needed. The gaps between true and estimated limits indicate the data distribution deviates somewhat from the assumed log-normal although the statistical test fails to reject the log-normal distribution (*p* = 0.4). RI: reference interval. CI: confidence interval

In some cases, a Gaussian distribution can be achieved by transforming the data using logarithmic, power function (Box-Cox), square root or other suitable transforms [25, 27]. Limits and their confidence intervals derived from transformed data are back-transformed before use. Required sample sizes are greater if data need to be transformed [27] (Fig. 3).

If reference limits are defined parametrically as mean ± 1.96 standard deviations when the data do not have a Gaussian distribution either before or after transformation, systematic misclassification will occur: although the parametric reference interval may enclose 95% of reference values, it will not be the central 95%. For example, amplitude data are usually skewed (Fig. 3). Parametric reference limits will misclassify, for example, ERGs with low amplitudes as normal, and misclassify ERGs with large amplitudes as supranormal (or hypernormal).

### Bootstrapping techniques

Bootstrapping is useful in deriving reference intervals because it allows inference about a population, e.g. its distribution, from a sample. By repeatedly resampling a dataset (with replacement), multiple, new, resampled datasets are generated in which original values may occur more than once, once, or not at all: the resampled datasets will emulate the results of repeating experiments. From these resampled datasets, bootstrap estimates of the reference limits and their precision (90% CIs) can be calculated. This technique improves the precision of reference limit estimation, so that the requirement for the 90% CI of a reference limit to be < 0.2 of the reference interval is achieved with smaller sample sizes than with the nonparametric technique [30]. The bootstrapping technique is suitable for data which is not, and cannot be transformed to be, Gaussian in distribution, and can be employed for relatively small samples (*n* ~ 40) [29, 31–33].

### Outliers

Measurements obtained from the reference sample group are curated to remove outlying data points [34]. There is a trade-off between removing outliers, which narrows the reference interval and thereby highlights more diseased cases, and removing useful data which thereby flags more normal cases. The emphasis is always on retaining data.

Inspection of graphed data is a helpful process, and data points distinctively separated from neighbouring points are examined to establish whether they are due to measurement error, operator or device error, subject compliance, deviations from protocol, or non-adherence to inclusion/exclusion criteria. Relying on intuitive insight from graphs should be used with caution; for example, the rightmost point in Figs. 1 and 3 was a sample from the underlying distribution and therefore should not be classified as an outlier.

Objective techniques exist to remove any remaining outlying data from a near-Gaussian distribution (before or after transformation), for example Pierce’s criterion, Grubbs' test, and Reed/Dixon's Q test [35]. Where the shape of the reference distribution is not known, Tukey's fences rejects outliers using nonparametric techniques [36]. Advanced numerical techniques have also been described [37]. Using Tukey’s far outliers (three interquartile ranges from the upper or lower quartile), for example, rejects two values per million from a Gaussian distribution but two values per thousand from the gamma distribution used in Figs. 1 and 3.

Outlier detection should be performed after any adjustments to the data are made (see sections on *Subject age *(below), and transformations in the *Parametric method* section (above)). For example, if peak times increase with age and if outlier detection is performed before adjusting values based on age, elderly (and very young) subjects may erroneously be more likely to be classified as outliers.

Prevention of outliers affecting estimates of age dependence or parametric method fit parameters may be done with robust fitting techniques [2, 32, 38]. Tukey’s biweights, instead of minimising the squared errors between the fit and the data, perform the minimisation iteratively after attenuating errors that are excessively large. Trimmed means or Tukey’s biweights can be used to estimate the mean, and the interquartile range can be used in place of standard deviation.

### Recommended number of subjects

While no single recommended number exists, a justified target is at least 120 subjects after outlier removal [26]. It is always better to have more subjects than fewer; larger numbers of subjects reduce the uncertainty of the reference limits and also enable finer-grained partitioning which may give tighter reference intervals, making it more likely that diseased subjects will be flagged to the clinician.

The key criterion for required sample size is that the precision with which the reference limits are known (their 90% confidence intervals or CIs) is small relative to the biological dispersion, i.e. the reference interval itself (Fig. 3). It is recommended that the CI of a reference limit should be < 0.2 of the whole reference interval [2, 27, 29]. CIs indicate the reliability of reference limits and therefore whether a test is able to meet clinical expectations. Meeting this criterion, especially for data at the long-tailed end of highly skewed distributions, may be difficult to achieve as the required sample size may be considerably beyond 120 per partition when using nonparametric methods. If the reference distribution is Gaussian, meeting this criterion may require as few as 55 subjects per partition [39].

In some instances, for example very young or highly myopic subjects, it may not be possible to collect sufficient reference data points. Recommendations for handling small reference datasets have been developed [40]. For sample sizes ≥ 20 but < 40, robust or parametric (if appropriate) techniques should be used; calculation of 90% CIs should only be undertaken to illustrate the magnitude of uncertainty, not for clinical classification as ‘indeterminate’. Data should be presented as a histogram with median (or mean) and minimum and maximum values stated. For sample sizes ≥ 10 but < 20, values should be listed in a ranked table with only the median (or mean) calculated [39]. It is not recommended that reference data from 10 or fewer subjects be reported, and subject-based reference intervals should be considered if so few reference subjects are available [40].

### Correlation between eyes

**Fig. 4:**
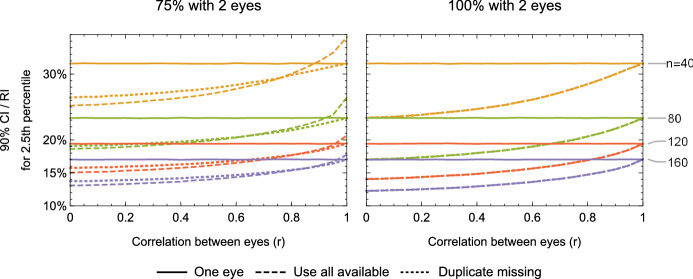
Uncertainty of reference limits, expressed as the ratio (%) of the 90% confidence interval (CI) of a limit to the whole reference interval (RI), as a function of inter-eye correlation. Three methods for handling correlation between eyes are shown: use one eye per subject (solid lines); use all available eyes as independent samples (dashed lines); and, for subjects with data from only one eye, duplicate the point so that all subjects have data from two eyes, then use all eyes as independent samples (dotted lines). Right panel: all subjects have results from both eyes. Left panel: 75% of subjects have results from both eyes and 25% have results only for one eye. For each correlation coefficient and number of subjects, samples of correlated Gaussian random variables were taken and the lower reference limit was estimated using the nonparametric method. The process was repeated 1,000,000 times for each condition. n: number of subjects

ERG measurements between the right and left eyes are correlated [18, 19, 41]. When estimating a reference limit, there are several acceptable strategies to compensate for inter-eye correlation. Data from only one eye per subject may be used, although because the inter-eye correlation is not perfect, information is lost. Averaging results between eyes is not recommended, as it erroneously reduces the effect of variability due to recording factors such as electrode placement: no such reduction in variability will occur during patient testing. If all reference subjects provide data from both eyes, using both eyes’ data will not affect the expected values of the reference limits but will improve their accuracy. In the limit of no correlation, using both eyes’ data is the same as doubling the sample size, as seen in the right panel of Fig. 4, where no correlation (*r* = 0) provides the same performance as doubling the number of subjects. In the limit of perfect correlation, using both eyes’ data has no effect: for example, the 10th percentile of the digits 0–9 is 1 no matter how many sets of those digits are used (and also can be seen at the rightmost side of plots in the right panel in Fig. 4). If only some subjects have data from both eyes, strategies to use all available information become more complex. Through simulations (Fig. 4), we found that duplicating single eye data from subjects where only one eye was tested (making perfectly correlated two-eye subjects), so that all subjects have a pair of results, works well across sample sizes and levels of correlation. The duplication method is never worse than the using only one eye and is better when the eyes are not perfectly correlated.

When estimating the uncertainty in reference limits, one must also compensate for inter-eye correlation, unless only data from one eye per subject were used. We found through simulations that bootstrapping subjects (not eyes) eliminate overly narrow confidence intervals resulting from having ‘duplicates’ in cases of high correlation between eyes, while also not affecting the confidence intervals in low correlation cases. Using generalised estimating equations [42], which estimate the correlation as a fit parameter, may also be useful in computing the confidence intervals.

### One or two reference limits?

Typically, pathology affects electrophysiological measures by reducing amplitude and increasing peak times, and it has been considered that one-tailed limits are suitable for evoked potential measures, i.e. that an evoked potential can only be too small or too late [43]. However, in clinical visual electrophysiology, findings in several pathologies contradict this. For example, early pattern VEP P100 peak are seen in some patients with visual pathway dysfunction [44, 45] and supranormal VEP amplitudes are also seen in certain conditions [46]. Whilst supranormal (or hypernormal) full-field ERG amplitudes have been related to pathology [47], the prevalence of extreme amplitudes (104 out of 5000 cases) may be that expected by chance [48]. For these reasons, the choice of constructing one- or two-sided reference limits should be made for each parameter based on the likelihood of too-early peak times or too-large amplitudes being seen in pathological cases. Where both extremes of a parameter are associated with pathology, the reference interval is from the central portion of the distribution, e.g. 2.5th to the 97.5th percentile. Where only one extreme is associated with pathology, the reference interval is the upper or lower portion of the distribution, and the single reference limit is the 5th or the 95th percentile as appropriate. When using one-sided reference limits, we propose still expressing its uncertainty as the ratio of its 90% CI to the two-tailed 95% reference interval (2.5th–97.5th percentile) rather than a one-tailed reference interval. The two-tailed reference interval is numerically more favourable than using either of the one-tailed reference intervals (5th–100th or 0th–95th percentile), which depends on the most extreme value measured and therefore does not converge with increasing *n*.

### Partitioning

The effect of demographic variables, such as gender, race or age (see Table 1), on any visual electrophysiological parameter can be gathered from the literature. Where a demographic affects a parameter such that there is both a statistically significant and a clinically meaningful difference between subgroup average values, partitions are made to create separate subgroups. One rule of thumb suggests separate reference ranges are not required unless subgroup averages differ by > 25% of the 95% reference range of the combined group [49]; a more stringent requirement of ~ 15% has also been suggested [50]. An alternative metric requires separate partitions if > 4% of reference data points from one subclass fall outside the reference limits for all groups combined [50]. A further recommendation requires separate subgroup reference ranges if the ratio of subgroup standard deviations is 1.5 or greater, regardless of any difference in subgroup means [50]. Given the challenges of recruiting and testing sufficient subjects per partition, limiting the number of partitions is advisable.

### Subject age

Unlike some demographic variables, age is a continuous value. Partitioning age into decades or some other grouping leads to artefacts at the group boundaries, where identical test results on the day before and the day of a subject’s birthday may switch classification from abnormal to normal (or vice versa) as the subject ages into a new age partition. Having more age groups reduces the changes in reference interval between adjacent groups, but requires more reference subjects.

The majority of visual electrophysiology parameters change during infancy and childhood, and to a lesser extent, in the elderly. For example, the P100 of the pattern reversal VEP is strongly dependent on age over the first year of life, being slower in younger babies: pooling infants and toddlers together in a reference dataset will create reference intervals which are too wide to detect abnormalities in toddler-aged patients [51]. Studies of age-related changes generally employ a cross-sectional study design where each reference subject provides data at a single age, with ages suitably sampled for robust centile estimation [52–54]. Given the onerous nature of testing small children, smaller sample sizes are likely per age group, which makes estimates vulnerable to extreme observations; optimal reference sample groups may require as many as 500 reference subjects [55].

Compensating for age with a continuous function (e.g. linear correction) may be preferable as it keeps all the subjects in the same partition. Robust curve fitting is useful in this process so that age compensation can happen before outlier removal [56, 57]. With many subjects in the reference distribution, the upper and lower reference limits can be separately fitted so that the width of the reference interval can change with age as well.

## Establishing reference intervals: indirect sampling

Where direct sampling of a reference population is not possible, reference intervals can be derived from patient data [58, 59], referred to as indirect sampling. Since patients often undergo visual electrophysiology tests to have a disease excluded, many do indeed have normal test results. It is therefore possible to extract an estimated ‘health-related’ sub-population from patient databases, although reference intervals derived this way may not reflect the general population [60, 61]. Discussion of indirect sampling techniques is beyond the scope of this work, but readers are referred to techniques described elsewhere, based on removal of outliers, systematic removal of subjects with certain clinical factors [62], removal of repeat measures, and statistical derivation of two sub-samples, one of which aims to reflect a ‘health-related’ sub-population [61, 63–65]. Data mining applications make such analyses of large datasets feasible [66–68].

## Transference of a reference interval

Establishing reliable reference intervals is time-consuming and costly. Where possible, reference intervals already established elsewhere should be used, providing quality conditions can be met. Transference of a reference value is the process of adapting a previously established reference interval to a new or updated test technique or test centre [2, 32, 69]. As an example, a centre previously established an ERG reference interval based on the 2004 Standard [70] using a 2.0 cd·s·m^−2^ flash, but wished to update their ERG test protocol to comply with the current stipulation of 3.0 cd·s·m^−2^ [12]. As another example, one may want to transfer reference data taken with one electrode type to another electrode type.

**Fig. 5:**
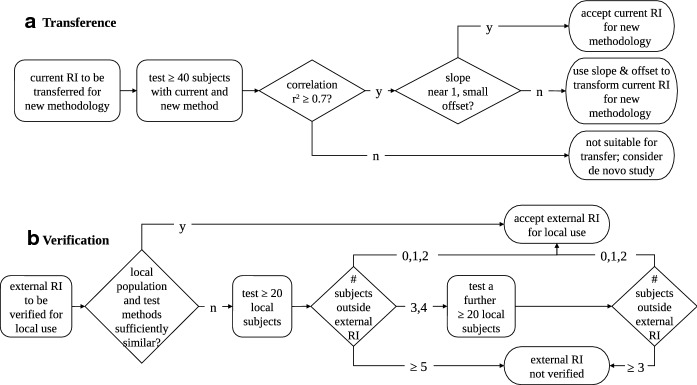
Flowcharts outlining simplified processes of **a** transference [71] and **b** verification [2]. RI reference interval, y yes, n no, # number of

Transference of a reference interval involves comparing results from the same subjects tested with both methods, which is the subject of an international guideline in clinical laboratories [2, 71] (Fig. 5). Measure at least 40 subjects using both the old and new test methods. If the results have high correlation (${r}^{2}\ge 0.7$) [72], a slope near one, and small offset, existing reference intervals can be used with the new test method. If the correlation is high, but the slope or offset are clinically significant, reference limits can be mathematically adjusted using values from the correlation equation. The measurements should span a wide range, and the magnitude of any offset (intercept) should be small relative to the data range and to the reference interval. Both diseased subjects and subjects free from disease can be used.

If a centre wishes to adopt a reference interval established elsewhere, visual electrophysiology has a great advantage over clinical laboratories, as the establishment of ISCEV standards has produced tightly defined stimulus, acquisition and analysis parameters, which results in very low intra-individual variation, as established for the pattern VEP [73] and the full-field ERG [74], even when different equipments are used. This greatly increases confidence in the possibility of transferring reference intervals.

## Verification (or validation) of a reference interval

Verification of a reference value is the process of ensuring that a reference interval established elsewhere can be adopted locally with reasonable confidence [2, 69, 72] (Fig. 5). This might typically occur when a centre wishes to use a manufacturer’s own, built-in reference data or another centre’s reference data. It should also be undertaken as part of transference of reference intervals.

Initial verification entails documented assessment of the original reference dataset, i.e. demographic variables and method of estimating the reference limits, and of the original test procedures: if these factors are subjectively judged to be comparable with the adopting centre’s test methods and patient population, then adoption is validated.

Further verification may be necessary, particularly if not all required details of the reference interval are available. The adopting centre recruits 20 local reference subjects who satisfy exclusion and partition criteria: if no more than two reference data points fall outside the primary reference interval, that interval can be considered acceptable for local use. If three or four data points fall outside the primary reference range, a further 20 local subjects should be recruited and tested; if no more than two reference data points from this second local sample group fall outside the primary reference interval, the interval can be considered acceptable for local use. Otherwise, a re-examination of test protocols should be considered, along with the possibility that the local patient population is substantially different to the reference subjects contributing to the primary reference sample.

This simple check is vulnerable to error for skewed distributions or variance differences between primary and local samples. If the full primary reference dataset is available, comparisons using Mann–Whitney U, Siegel–Tukey or Kolmogorov–Smirnov are more sensitive and specific [2]. For greater accuracy in deciding the acceptability of a primary reference dataset, for example where there is a particular local need for accuracy, larger numbers of local reference subjects can be tested [71].

## Interpreting serial measurements: subject-based reference values

Population-based reference intervals, as discussed so far, are primarily used for a single, diagnostic assessment, for case-finding, and for screening. However, their high inter-individual variability means they may not be sensitive to changes within a patient over time: an individual could show significant worsening of a parameter even though it remains well within the reference interval [41, 75, 76]. In some developed economies, healthcare is increasingly devoted to management of disease, with proliferation of serial measurements on patients. In such cases, subject-based reference values from longitudinal data may be more useful than cross-sectional population-based reference values to decide whether a parameter has changed by a clinically meaningful amount—the ‘delta check’. The size of the change should exceed that expected to be due to inherent sources of variability such as acquisition or stimulus changes, electrode positioning, and to the individual’s biological changes, some of which can be minimised by standardised protocols related to time of day, pupil diameter and so forth.

The critical change size (or critical difference) is termed the repeatability coefficient (RC) [77] and is described as:

3 $$RC = \pm z {\text{CV}}\sqrt 2 \;{\text{or}}\;RC = \pm z {\text{SD}}\sqrt 2$$

where* z* is the* z*-statistic, CV is the coefficient of variation of replicates and SD is the standard deviation of replicates. Generally, standard deviations should be used for times (variability expressed in ms), while CVs should be used for amplitudes (variability expressed in percent changes). The* z*-statistic is conventionally taken to be 1.96, giving a 5% probability of a false positive. Larger *z* values increase the size of the change required to be classified as a significant change (the RC), thus decreasing the false-positive rate while increasing the false-negative rate [78]. If *z* = 1.96, Eq. 3 simplifies to RC = 2.77 × CV or RC = 2.77 × SD. With data from multiple subjects, each with the same number of replicates, the average CV or SD is used. If the number of replicates differs between subjects, a weighted average is used to account for the greater certainty of the precision in subjects with more replicates:

4 $${\text{SD}} = \sqrt {\frac{{\mathop \sum \nolimits_{i = 1}^{N} \left( {k_{i} - 1} \right)s_{i}^{2} }}{{\mathop \sum \nolimits_{i = 1}^{N} (k_{i} - 1 )}}}$$

where ${k}_{i}$ is the number of replicates for the $i$th subject, ${s}_{i}$ is the standard deviation for the $i$th subject, and $N$ is the total number of subjects. The CV is defined analogously. When computing the RC, data from each eye should not be combined but treated as separate ‘subjects’. Combining data from both eyes in calculating a standard deviation will artificially increase the standard deviation in cases where expected value of the two eyes is not the same (e.g. unilateral disease). See simulations in the supplementary material for additional evidence for treating each eye separately. Uncertainty of the RC can be computed, for example, by using bootstrapping as described.

The RC can be measured with as few as eight subjects [79]. Clinically meaningful flash VEP changes [80] and ERG changes have been established for patients with [41, 81–84] and without retinal disease [81, 85]; RCs established from stable but diseased patients may sometimes be appropriate.

## Multidimensional reference region

Visual electrophysiology data are naturally bi-variate (e.g. the pattern ERG P50 has both an amplitude and a peak time) with both data portions being related to some degree, being derived from the same part of the organ system. Multiple further related measures are often captured at the same recording (e.g. pattern ERG N95 parameters) and even more during a test session, which may also record full-field ERGs, multifocal ERGs and other indicated tests. They therefore naturally lend themselves to multivariate reference regions rather than multiple univariate reference intervals as have been discussed so far, thereby reducing the risk of false-positive findings. Despite their suitability for scoring multiple tests assessing the same organ system, multivariate reference regions are only slowly gaining traction as a diagnostic tool [86–88], perhaps because of difficulties with clinical interpretation or the relatively complex maths required [89, 90].

## Clinical interpretation

### Relation between reference intervals, clinical decision limits and disease detection

Measurements falling within the reference interval are consistent with the reference population, i.e. people with normal vision, and are classified as normal. Normal measurements do not guarantee the patient is disease-free; for example, the patient may have a disease that does not affect that measurement. Measurements outside the reference interval are not consistent with the reference population and are classified as abnormal or atypical. Patients with atypical results can be examined more closely or more frequently with more concern for cases where the results are far from the reference limits in a direction associated with disease. Reference limits cannot be used to tell which disease a patient might have, but they can highlight cases where some disease is suspected.

Measurements falling within the CI of either the upper or lower reference limit may be considered ‘indeterminate’ [91] to some extent. For small reference samples, many patient parameters will fall in these indeterminate zones. No clear guidance exists on how to handle this, and it may simply be advisable to be aware of the size of reference limits’ CIs when reporting and interpreting clinical visual electrophysiology recordings.

For serial (or longitudinal) testing, a measurement outside the repeatability coefficient (RC) indicates that patient’s result has changed, either improving or worsening depending on what is known about the way a particular disease affects the measurement.

Clinical decision limits, by contrast, classify patients as diseased or healthy. They are determined using data from diseased subjects as well as healthy subjects and consider the balance of test sensitivity and specificity. For example, the World Health Organization recommends a clinical decision limit of glycated haemoglobin (HbA1c) ≥ 6.5% to classify patients as having diabetes [92].

If a 95% reference interval is used as a clinical decision limit for detecting a disease, the test specificity (probability a healthy subject is classified as healthy) is 95%, because that is the proportion of reference subjects enclosed by the 95% reference interval. Reference intervals cannot be used to classify a patient as having a particular disease because the disease’s influence on the measurement is not used when constructing the reference intervals (in fact, no diseased subjects are used in making reference intervals). In other words, 95% reference intervals used as clinical decision limits have no impact on test sensitivity (probability a diseased subject is classified as diseased).

### Adjusting for multiple measurements

The use of a 95% reference range means that any single parameter has a one in 20 chance of being classified as abnormal when no abnormality exits, so reporting multiple parameters from multiple tests (*n* parameters total) carries an increasing risk (1–0.95^*n*^) [20] of false-positive findings (if all *n* parameters are uncorrelated with each other) and may require to be adjusted for simultaneous statistical inference [19]. Useful test interpretation, following factual classification of each parameter can utilise understanding of the origin and interaction between parameters to mitigate such risks. For example, a borderline-small ERG a-wave may be of concern in the face of an abnormally delayed a-wave peak time or an abnormally small b-wave, but would be of less concern if all related parameters are normal.

## Conclusions

Clinical visual electrophysiology has long established and highly standardised tests, which appear to have low within-subject variability. Current ISCEV standards indicate that each centre should establish its own reference data; however, undertaking this process adequately is onerous and likely not to be feasible for all centres. Transferring and verifying reference datasets from elsewhere, with due care to quality measures, offer the possibility of sharing high-quality, large reference datasets. It also allows high-quality legacy reference data to continue to be used even when standards are updated. Such initiatives have successfully been undertaken in other clinical areas with the goal of harmonising reference limits, to the great benefit of patients [93, 94].

Clinical electrophysiology has advantages over imaging techniques, because of its consistency due to international standards [9–13] and because of its generation of objective, quantitative data that can be robustly classified using reference data. This paper describes methods of creating reference limits either by establishing them de novo or by transferring or validating limits acquired elsewhere. Our emphasis has been on clinical electrophysiology of vision; however, these methods are also valid for other quantitative clinical measurements on bilateral systems where intra-subject correlation needs to be considered. Good quality reference limits minimise false-positive and false-negative results, thereby maximising the clinical utility and patient benefit. Quality indicators include using appropriately sized reference datasets with appropriate numerical handling for reporting; using subject-based reference limits where appropriate; and limiting tests for each patient to only those which are clinically indicated, independent and highly discriminating.

## Supplementary Information

Below is the link to the electronic supplementary material.

Supplementary file1 (PDF 256 KB)

## Abbreviations

ERG: Electroretinogram
VEP: Visual evoked potential
EOG: Electrooculogram
ISCEV: International Society for Clinical Electrophysiology of Vision
CI: Confidence interval
RI: Reference interval
RC: Repeatability coefficient
CV: Coefficient of variation
SD: Standard deviation

## Footnotes

1 The term ‘normal’ (or ‘normative’) is obsolete because of lack of scientific clarity due to its triple meaning in the English language (clinically healthy; statistically Gaussian; popularly connoting conventional). There also is flawed circular logic inherent in equating a ‘normal’ person with a person free from disease, while disease is diagnosed based on measurable characteristics of ‘normal’ individuals. Finally, the implication that an individual is ‘abnormal’ should a measurement lie outside certain limits is pejorative [1].

2 Percentiles are derived from a sample, as estimates of true population percentiles, by calculating an index position of the ranked sample data or using linear extrapolation for non-integer indices. Index = 0.5 + *p n*, where *p* is the percentile of interest and *n* the sample size [23] has higher accuracy than alternative index calculations [24].

## Declarations

### Human and animals welfare

The study involved no research on animals.
