# Supplementary material for: Reference ranges for clinical electrophysiology of vision
Source: Doc Ophthalmol. 2021 Apr 21;143(2):155–70. doi: 10.1007/s10633-021-09831-1 (PMC8494724; doi:10.1007/s10633-021-09831-1)
Supplement: Supplementary file 1 — Supplementary file1 (PDF 256 KB) [file 10633_2021_9831_MOESM1_ESM.pdf]

Reference ranges for clinical electrophysiology of vision

C.Quentin Davis, Ruth Hamilton

29 Jan 2021

This document is the source code for the simulation studies performed in the paper, as well as the source for the figures. Run-time is about 1 day.

Figures 1 and 3

Underlying distribution

A gamma distribution is used. The fixed measurement list came from one call of RandomSample. Setting use-FixedMeasurementQ to False will draw a new sample.

In[446]:=
```
sampleDist = GammaDistribution[3, 10, 1, 10];
useFixedMeasurementsQ = True;
(* generated by RandomSample *)
fixedMeasurements = measurements =
   {22.153044209662724`, 45.063419889890675`, 24.864945693646426`, 19.49003642953529`,
    40.869674712711316`, 54.97254116324288`, 41.58682282381315`, 25.246595314846644`,
    34.83537902123453`, 30.114859745003372`, 16.186686518677636`, 87.78140230671713`,
    50.6826490628012`, 34.6539077196976 4`, 51.612198485706784`, 36.7661047853349`,
    29.90993768323045`, 56.7556060361014`, 40.66613528373235`, 19.43661032430817`,
    23.677423305806645`, 29.62936911076724`, 33.6509395895 6017`, 96.12268379604969`,
    22.478752358048744`, 24.556426731533705`, 29.502327636646555`, 39.54944494503458`,
    45.767654657641714`, 25.6893805842655`, 42.0296808138411`, 48.97183595787073`,
    24.683351726538106`, 87.35032096220267`, 29.679713173119303`, 28.927467471783224`,
    24.428673190494553`, 48.35281004041458`, 49.102816187873174`, 68.68199605738519`,
    15.173709192193565`, 65.8336165880547`, 16.976252998743295`, 40.643956557873885`,
    45.35485890341419`, 24.89335818694829`, 30.548882685503038`, 21.33153034659176`,
    51.20756364201937`, 25.563715853333175`, 23.91463554108916`, 71.56777667056069`,
    77.01270618659598`, 18.08666164588262`, 23.332127168755726`, 59.387244096563094`,
    59.752549375217924`, 31.395626995847504`, 32.87064158143133`, 51.101946676717816`,
    44.505518577431566`, 17.523627032573167`, 11.981534036881014`, 76.54841508807186`,
    75.1692298615621`, 27.51541858250032 7`, 44.328405985178804`, 34.73599108153914`,
    23.58147688535602`, 35.81018446031785`, 43.23366445529434`, 21.67382878891903`,
    78.11657264722297`, 15.741182143650928`, 26.465650317404823`, 17.41475111186082`,
    35.85023665323233`, 50.143518588914176`, 46.86196549704007`, 33.44261163320559`,
    30.25220547609004`, 75.97134848589312`, 20.887823629309583`, 75.61002629971094`,
    36.74548500965296`, 26.079200553788578`, 36.451343122099885`, 50.22647856637654`,
    29.1450631808350 8`, 41.47111107669255`, 76.8209689156662`, 26.51080596704056`,
    43.59297032708701`, 32.83192080555825`, 59.15270617716227`, 31.52764321447861`,
    63.802573996816854`, 25.68003647384172`, 23.2345873063929`, 62.679878389904495`,
    58.47573761011871`, 12.989699689015087`, 26.120279060462003`, 15.385661462246423`,
    28.871005981116415`, 14.217359963711221`, 52.21472365841354`, 40.510409456157106`,
    28.11067651264225`, 23.402203925552037`, 46.80304970912407`, 58.47469573498205`,
    45.7406314735292`, 63.3626885183977`, 36.81631410085463`, 70.29269074913717`,
    46.20716144790259`, 39.80594914727843`, 43.802824830078066`, 71.674365786648`};
```

Analysis methods

```
(*,"Interpolation type B: pn+.5", "Hydrologist method"}};*)
interpolationType = {{1/2, 0}, {0, 1}};

(* fits Gaussian to data, returns 5th, 50th, and 95th percentile for the desired quantile. *)
gaussianMethod[data_, quant_] := Module[{μ, σ, n, α = .1, Zp = -√2 InverseErfc[2 quant]},
  μ = Mean@data; σ = StandardDeviation@data; n = Length@data;
  (* 90% CI from Equation 2 in paper *)
  {Quantile[NormalDistribution[μ, σ], quant] - 2.81 σ / Sqrt[Length@data]
   , Quantile[NormalDistribution[μ, σ], quant]
   , Quantile[NormalDistribution[μ, σ], quant] + 2.81 σ / Sqrt[Length@data]
  }
 ];
(* fits Gaussian to data, returns , returns 5th,
50th, and 95th percentile for the desired quantile.
   Computation takes > 1 minute, so memoize the answer *)
```

```
gaussianMethodExact[data_, quant_] :=
 gaussianMethodExact[data, quant] = Module[{μ, σ, n, α = .1, Zp = -√2 InverseErfc[2 quant] },
   μ = Mean@data; σ = StandardDeviation@data; n = Length@data;
   (* Lawless (2002), as cited in Chakraborti (2007) equation 10*)
   {μ - Quantile[NoncentralStudentTDistribution[n - 1, -Sqrt[n] Zp], 1 - α / 2] σ / Sqrt@n,
    Quantile[NormalDistribution[μ, σ], quant],
    μ - Quantile[NoncentralStudentTDistribution[n - 1, -Sqrt[n] Zp], α / 2] σ / Sqrt@n
   }
  ];
(* same as gaussian method after transforming the data*)
logNormalMethod[data_, quant_] := Exp[gaussianMethod[Log /@ data, quant]];

(* fits Gaussian to data, returns 5th, 50th, and 95th percentile for the desired quantile *)
gaussianBootMethod[data_, quant_] := Module[{μ, σ, boot, d},
   boot = ParallelTable[
     d = RandomChoice[data, Length@data]; μ = Mean@d; σ = StandardDeviation@d;
     Quantile[NormalDistribution[μ, σ], quant]
     , 10^4
    ];
   {Quantile[boot, .05, interpolationType],
    Quantile[boot, .5, interpolationType], Quantile[boot, .95, interpolationType]}
  ];

(* bootstrap, returns 5th, 50th, and 95th percentiles for the desired quantile *)
bootMethod[data_, quant_] := Module[{boot},
   boot =
    ParallelTable[Quantile[RandomChoice[data, Length@data], quant, interpolationType], 10^3];
   {Quantile[boot, .05, interpolationType], Quantile[boot, .5, interpolationType],
    Quantile[boot, .95, interpolationType]}

  ];

(* Rank method, returns 5th, 50th, and 95th percentiles for the desired quantile *)
(* Ranks from Table 8 of CLSI (2010)EP28-A3C Defininining, establishing,
and verifying reference intervals in the clinical laboratory *)
rankMethod[data_, quant_] := Module[{len, lower, upper, sortedData},
   len = Length@data; sortedData = Sort@data;

                    ⎧ 1  len < 188
                    ⎪ 2  len < 250
                    ⎪ 3  len < 308
   lower[len_] :=   ⎨ 4  len < 364;
                    ⎪ 5  len < 418
                    ⎪ 6  len < 471
                    ⎩ 7  len < 523
```

```
                  ⎡ 7   len < 133
                  ⎢ 8   len < 161
                  ⎢ 9   len < 190
                  ⎢ 10  len < 219
                  ⎢ 11  len < 249
                  ⎢ 12  len < 280
  upper[len_] := ⎨ 13  len < 310;
                  ⎢ 14  len < 341
                  ⎢ 15  len < 373
                  ⎢ 16  len < 404
                  ⎢ 17  len < 436
                  ⎢ 18  len < 469
                  ⎣ 19  len < 501

  If[quant < .5, {sortedData[[lower[len]]],
     Quantile[data, quant, interpolationType], sortedData[[upper[len]]]}
   , {sortedData[[-upper[len]]], Quantile[data, quant, interpolationType],
     sortedData[[-lower[len]]]}
  ]
 ];
```

Figures for Gamma distribution

In[456]:=

```
halfY = Maximize[PDF[sampleDist, xxx], xxx][[1]] / 2;
isize = {Automatic, 320 / GoldenRatio};
xmax = 10 + Round@Max@measurements;
xmin = -12;

fig3plotter[method_, label_, panelLabel_, xAxisLabel_, yAxisLabel_] := Module[{},
   minFit = Quiet@method[measurements, .025];
   maxFit = Quiet@method[measurements, .975];
   yBox = .88;
   Overlay[{ListPlot[Flatten[Table[
         MapIndexed[{#1, First[#2]} &, ConstantArray[(histList[[1, i]] + histList[[1, i + 1]]) / 2,
           histList[[2, i]]]], {i, Length@histList[[2]]}], 1]
      , ImageSize → isize, ImagePadding → {{Automatic, Automatic}, {20, 0}},
      Axes → False,
      Frame → {True, True, False, False}, PlotStyle → PointSize[.018],
      FrameLabel → {xAxisLabel, yAxisLabel}, FrameTicks → None,
      PlotRange → {{xmin, xmax}, Automatic}, LabelStyle → Directive[Black, 12]
      , PlotLabel → label
      , Epilog → {Opacity[.2], Rectangle[{minFit[[1]], 0}, {minFit[[3]], 100}]
         , Rectangle[{maxFit[[1]], 0}, {maxFit[[3]], 100}]
         , Arrowheads[{-.03, .03}],
         Opacity[1], Red
         , Inset[Framed[Style[StringForm["CI =\n`` × RI",
              Round[(-Subtract @@ maxFit[[{1, 3}]]) / (maxFit[[2]] - minFit[[2]]), .01]], 12]
            , Background → Directive[Opacity[.7], White], RoundingRadius → 5, FrameStyle → Gray],
           {Mean@maxFit[[{1, 3}]], yBox Max@histList[[2]]}, {Center, Center}]
         , Inset[Framed[Style[StringForm["CI =\n`` × RI", Round[
               (-Subtract @@ minFit[[{1, 3}]]) / (maxFit[[2]] - minFit[[2]]), .01]], 12]
            , Background → Directive[Opacity[.7], White], RoundingRadius → 5, FrameStyle → Gray],
           {Mean@minFit[[{1, 3}]], yBox Max@histList[[2]]}, {Center, Center}]
```

```
          , Opacity[1], Arrow[{{minFit[[1]], yBox Max@histList[[2]]},
            {minFit[[3]], yBox Max@histList[[2]]}}]
          , Opacity[1], Arrow[{{maxFit[[1]], yBox Max@histList[[2]]},
            {maxFit[[3]], yBox Max@histList[[2]]}}]
          , Black
          , actSign = If[Quantile[sampleDist, .975] < maxFit[[2]], 1, -1];
          Inset[Rotate[Style["true", 12], actSign 90 Degree], {Quantile[sampleDist, .975] - actSign,
            .68 Max@histList[[2]]}, {If[actSign == 1, Right, Left], Top}]
          , Line[{{Quantile[sampleDist, .025], 0 Max@histList[[2]]},
            {Quantile[sampleDist, .025], .68 Max@histList[[2]]}}]
          , Line[{{Quantile[sampleDist, .975], 0 Max@histList[[2]]},
            {Quantile[sampleDist, .975], .68 Max@histList[[2]]}}]
          , Red,
          Line[{{maxFit[[2]], 0 Max@histList[[2]]}, {maxFit[[2]], .68 Max@histList[[2]]}}]
          , Line[{{minFit[[2]], 0 Max@histList[[2]]}, {minFit[[2]], .68 Max@histList[[2]]}}]
          ,  Inset[Rotate[Style["estimate", 12], -1 actSign 90 Degree],
           {maxFit[[2]] + actSign, .68 Max@histList[[2]]}, {If[actSign == 1, Left, Right], Top}]
         }
       , GridLinesStyle → Directive[Black, Thickness[Medium]]
      ]
      , Text[Framed[Style[panelLabel, 18], RoundingRadius → 5, Background → LightBlue]]
     }
    ]
   ];
Table[
 measurements = If[useFixedMeasurementsQ, fixedMeasurements, RandomVariate[sampleDist, 120]];
 histList = HistogramList[measurements, {5}];
 {fig1 = Column[{
     Show[{
       Plot[PDF[sampleDist, x], {x, xmin, xmax}, PlotRange → (rng = {-.0005, .03}) ,
        ImageSize → isize, Axes → False,
        FrameTicks → None, Frame → {True, True, False, False},
        LabelStyle → Directive[Black, 12],
        FrameLabel → {(*"reference value"*)"", "frequency"}
        , GridLines → {{
           Quantile[sampleDist, .025](*,Quantile[sampleDist,.50]*), Quantile[sampleDist, .975]}
          , {0}}
        , GridLinesStyle → Black
        (*,PlotLabel→"Distribution from reference population\n(Idealized data)"*)
        , Epilog → {
          Inset[Style[Rotate["lower reference limit", 90 Degree], 12],
           {Quantile[sampleDist, .025] - 2, 2.1 halfY}, {Right, Top}]
          (*,Inset[Style[Rotate["median",90 Degree],12],
           {Quantile[sampleDist,.5]-1,halfY},{Right,Center}]*)
          , Inset[Style[Rotate["upper reference limit", 90 Degree], 12],
           {Quantile[sampleDist, .975] - 1, 2.1 halfY}, {Right, Top}]
          , Arrowheads[{-.05, .05}], Arrow[
           {{Quantile[sampleDist, .025], halfY / 10}, {Quantile[sampleDist, .975], halfY / 10}}]
          , Inset[Style["reference interval    ", Directive[12]],
           {50, halfY / 10}, {Center, Bottom}]
          , Inset[Style["2.5%", 12], pos = {(Quantile[sampleDist, .005] - xmin) / 200, halfY / 2},
           {Center, Bottom}], Line[{pos, {Quantile[sampleDist, .025 - .025 / 2],
             PDF[sampleDist, Quantile[sampleDist, .025 - .025 / 2]] / 2}}],
          , Inset[Style["95%", 12], {Quantile[sampleDist, .5], halfY / 2}, {Center, Bottom}]
          , Inset[Style["2.5%", 12],
```

```
          pos = {Quantile[sampleDist, .975] + (xmax - Quantile[sampleDist, .975]) / 2, halfY / 2},
          {Center, Bottom}], Line[{pos, {Quantile[sampleDist, .975 + .025 / 2],
            PDF[sampleDist, Quantile[sampleDist, .975 + .025 / 2]] / 2}}]
        }
      ]
      , Plot[PDF[sampleDist, x], {x, Quantile[sampleDist, .025], Quantile[sampleDist, .975]},
        PlotRange → rng, Filling → Axis, PlotStyle → Green]
      , Plot[PDF[sampleDist, x], {x, xmin, Quantile[sampleDist, .025]},
        PlotRange → rng, Filling → Axis, PlotStyle → Red]
      , Plot[PDF[sampleDist, x], {x, Quantile[sampleDist, .975], xmax},
        PlotRange → rng, Filling → Axis, PlotStyle → Red]
      , Plot[PDF[sampleDist, x], {x, xmin, xmax}, PlotRange → All]
     }]
    ,
    ListPlot[Flatten[Table[
       MapIndexed[{#1, First[#2]} &, ConstantArray[(histList[[1, i]] + histList[[1, i + 1]]) / 2,
         histList[[2, i]]]], {i, Length@histList[[2]]}], 1]
     ,
     ImageSize → isize, Frame → {True, True, False, False}, Axes → False,
     PlotRange → {{xmin, xmax}, Automatic}, PlotStyle → PointSize[.018],
     FrameLabel → {"reference value", "n"}, FrameTicks → None, LabelStyle → Directive[Black, 12]
     (*,PlotLabel→"Reference distribution\n(Measurements)"*)
    ]
   }],
  fig3 = Grid[{
    {
     fig3plotter[rankMethod, "non-parametric", "a", None, "n"]
     , fig3plotter[bootMethod, StringForm["non-parametric bootstrap"], "b", None, None]
    }
    , {
     fig3plotter[gaussianMethod, StringForm["parametric (Gaussian, p `` ``)",
       If[DistributionFitTest@measurements < .001, "<", "="],
       If[DistributionFitTest@measurements < .001, "0.001", NumberForm[DistributionFitTest[
          measurements, NormalDistribution[α, β]], 1]]], "c", "reference value", "n"]
     , ft = DistributionFitTest[measurements, LogNormalDistribution[μ, σ]];
     fig3plotter[logNormalMethod, StringForm["   parametric (log normal, p `` ``)", If[ft < .001,
        "<", "="], If[ft < .001, "0.001", NumberForm[ft, 1]]], "d", "reference value", None]
    }
   }]
 },
 1]
```

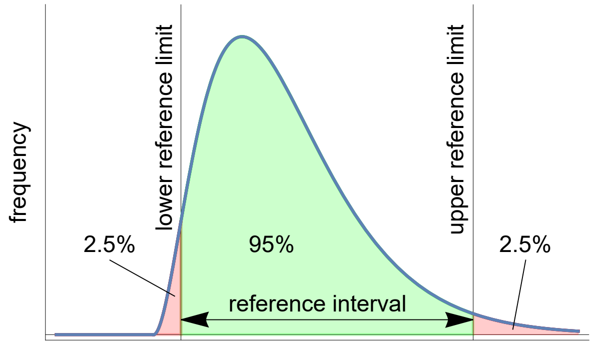

Out[461]= {{

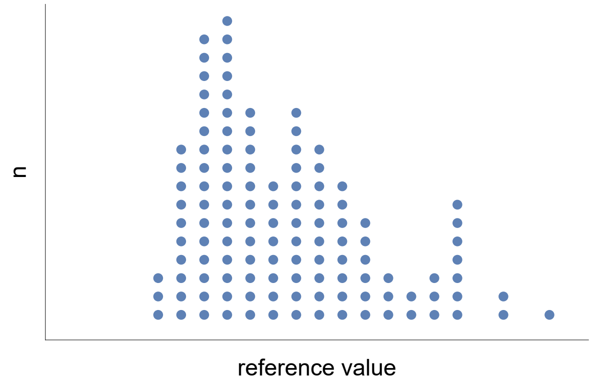

,

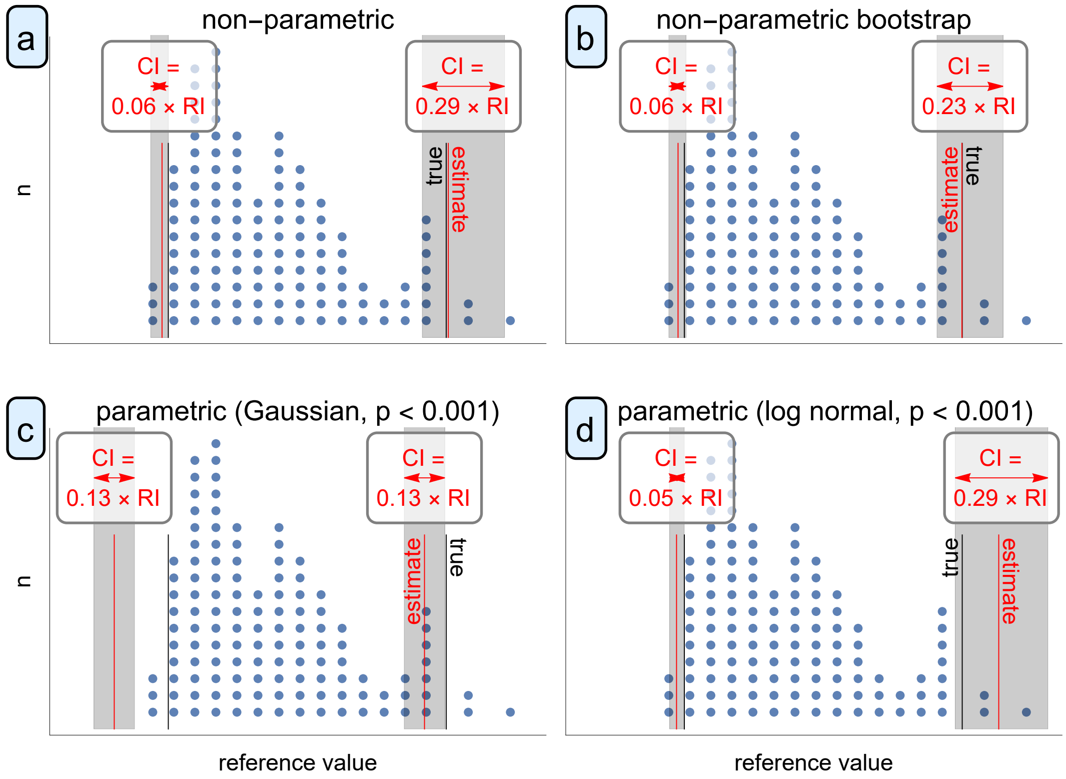

}}

In[462]:=
```
Export[StringTemplate["X:\\RIM\\science\\reference intervals\\figures\\Fig 1.``"][#],
    fig1] & /@ {"pdf", "eps"};
Export[StringTemplate["X:\\RIM\\science\\reference intervals\\figures\\Fig 3.``"][#],
    fig3] & /@ {"pdf", "eps"};
```

Fig 2: Gaussian confidence intervals

Compute all 4 limit points. The outer points fail to converge/evaluate in Mathematica for n > 165. Quantile doesn't need the pn+.5 parameter because this is an exact PDF, not samples.

In[464]:=
```
calcNoncentralError[n_] := calcNoncentralError[n] =
  With[{ Zp = 1.96, nZp = -1.96, α = .1},
   {n, Quantile[NoncentralStudentTDistribution[n - 1, -Sqrt[n] Zp], 1 - α / 2] / Sqrt[n] /
     (-Zp + 2.81 / Sqrt[n]),
    Quantile[NoncentralStudentTDistribution[n - 1, -Sqrt[n] Zp], α / 2] / Sqrt[n] /
     (-Zp - 2.81 / Sqrt[n]),
    Quantile[NoncentralStudentTDistribution[n - 1, -Sqrt[n] nZp], 1 - α / 2] / Sqrt[n] /
     (-nZp + 2.81 / Sqrt[n]),
    Quantile[NoncentralStudentTDistribution[n - 1, -Sqrt[n] nZp], α / 2] / Sqrt[n] /
     (-nZp - 2.81 / Sqrt[n])
   }]
```

In[465]:=
```
approxToNoncentralT =
 ParallelTable[calcNoncentralError[n], {n, {5, 6, 7, 8, 9, 10, 11, 12, 13, 14, 15, 16, 17,
    18, 19, 20, 25, 30, 35, 40, 50, 60, 80, 90, 100, 110, 120, 130, 140, 150, 155, 165}}]
```

Out[465]=
```
{{5, 1.50802, 1.52599, 1.52599, 1.50802}, {6, 1.37897, 1.3933, 1.3933, 1.37897},
 {7, 1.30222, 1.31356, 1.31356, 1.30222}, {8, 1.25133, 1.26051, 1.26051, 1.25133},
 {9, 1.21512, 1.22272, 1.22272, 1.21512}, {10, 1.18804, 1.19445, 1.19445, 1.18804},
 {11, 1.16702, 1.17252, 1.17252, 1.16702}, {12, 1.15023, 1.15502, 1.15502, 1.15023},
 {13, 1.13651, 1.14073, 1.14073, 1.13651}, {14, 1.12509, 1.12885, 1.12885, 1.12509},
 {15, 1.11543, 1.11881, 1.11881, 1.11543}, {16, 1.10716, 1.11022, 1.11022, 1.10716},
 {17, 1.09999, 1.10279, 1.10279, 1.09999}, {18, 1.09373, 1.09629, 1.09629, 1.09373},
 {19, 1.0882, 1.09057, 1.09057, 1.0882}, {20, 1.08329, 1.08548, 1.08548, 1.08329},
 {25, 1.06516, 1.06674, 1.06674, 1.06516}, {30, 1.05351, 1.05473, 1.05473, 1.05351},
 {35, 1.0454, 1.04638, 1.04638, 1.0454}, {40, 1.03942, 1.04025, 1.04025, 1.03942},
 {50, 1.0312, 1.03182, 1.03182, 1.0312}, {60, 1.02582, 1.02631, 1.02631, 1.02582},
 {80, 1.0192, 1.01955, 1.01955, 1.0192}, {90, 1.01702, 1.01732, 1.01732, 1.01702},
 {100, 1.01528, 1.01555, 1.01555, 1.01528}, {110, 1.01386, 1.01411, 1.01411, 1.01386},
 {120, 1.01269, 1.01291, 1.01291, 1.01269}, {130, 1.01169, 1.0119, 1.0119, 1.01169},
 {140, 1.01085, 1.01104, 1.01104, 1.01085}, {150, 1.01011, 1.01029, 1.01029, 1.01011},
 {155, 1.00978, 1.00995, 1.00995, 1.00978}, {165, 1.00918, 1.00934, 1.00934, 1.00918}}
```

The parallel table above breaks the memoize feature of the definition, so define them below without Parallel.

In[466]:=
```
Table[calcNoncentralError[approxToNoncentralT[[i, 1]]] = approxToNoncentralT[[i]],
  {i, Length@approxToNoncentralT}];
```

```
err[n_] := Interpolation[{#[[1]], 100 (#[[2]] - 1)} & /@ approxToNoncentralT[[All, 1 ;; 2]]][n];
fig2 = ListLogLogPlot[{
   {#[[1]], 100 (#[[2]] - 1)} & /@ approxToNoncentralT,
   {#[[1]], 100 (#[[3]] - 1)} & /@ approxToNoncentralT,
   {#[[1]], 100 (#[[4]] - 1)} & /@ approxToNoncentralT,
   {#[[1]], 100 (#[[5]] - 1)} & /@ approxToNoncentralT},
  (*PlotLegends→
   {"Lower CI for 2.5%","Upper CI for 2.5%","Lower CI for 97.5%","Upper CI for 97.5%"},*)
  Frame → {True, True, False, False}, Joined → True, FrameTicks →
   {{Automatic, None}, {Flatten[{{5, 10, 100}, Table[Round[n /. Quiet@Solve[ee == err[n], n][[1]]],
        {ee, {10, 5, 2, 1}}]
      , {#, ""} & /@ Range[10], {10 #, ""} & /@ Range[10], {100 #, ""} & /@ Range[10]
     }, 1], None}},
  Epilog → {Thin, Dotted, Table[Line[{Log@{n /. Quiet@Solve[ee == err[n], n][[1]], .1},
       Log@{n /. Quiet@Solve[ee == err[n], n][[1]], ee}, Log@{.1, ee}}], {ee, {10, 5, 2, 1}}]
   },
  LabelStyle → Directive[Black, 12], FrameLabel → {"n", "% error in Equation 2 limits"}
 ]
```

Out[468]=

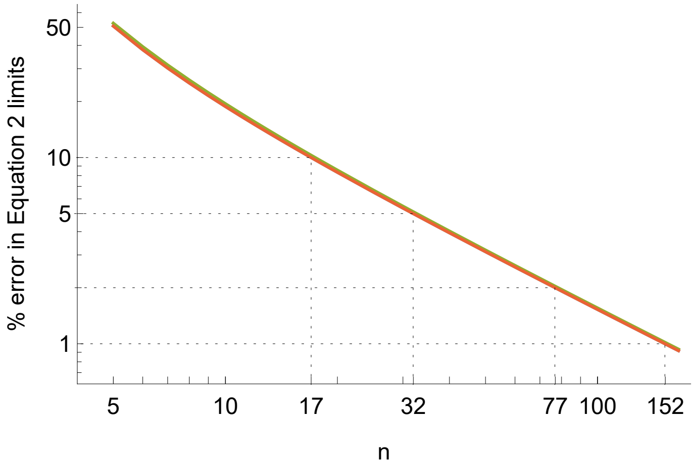


```
In[469]:= Export[StringTemplate["X:\\RIM\\science\\reference intervals\\figures\\Fig 2.``"][#],
     fig2] & /@ {"pdf", "eps"};
```

Fig 4: Correlation between eyes

Gaussian is symmetric, so picked the 2.5th percentile without loss of generality.

```
μ1 = 1 ;
μ2 = 1;
σ = 2;
Timing[rmsTwoEyes = Dataset@Flatten@ParallelTable[
      trueMean = μ1;
      trueSD = σ;
      percentile2p5 = InverseCDF[NormalDistribution[trueMean, trueSD], .025];
      referenceIntervalWidth =
       InverseCDF[NormalDistribution[trueMean, trueSD], .975] - percentile2p5;
      x1 = NormalDistribution[0, 1];
      x2 = NormalDistribution[0, 1];
      t = Table[
        rvx1 = RandomVariate[x1, n];
```

```
    rvx2 = RandomVariate[x2, n];
    y1 = μ1 + σ rvx1;
    y2 = μ2 + σ (ρ rvx1 + Sqrt[(1 - ρ^2)] rvx2);
    data = Join[Transpose[{y1[[1 ;; Floor[percentWithTwoEyes n]]],
        y2[[1 ;; Floor[percentWithTwoEyes n]]]}], y1[[Floor[percentWithTwoEyes n] + 1 ;; -1]]];
    duplicator = If[Length@# > 0, #, {#, #}] & /@ data;
    {Quantile[Flatten@data, .025, interpolationType] - percentile2p5
     , (*2*)Quantile[y1, .025, interpolationType] - percentile2p5
     , (*3*)Quantile[Flatten@duplicator, .025, interpolationType] - percentile2p5
     , (*4*)Mean@Flatten@data - trueMean
     , (*5*)Mean@y1 - trueMean
     , (*6*)Mean@Flatten@duplicator - trueMean

    }
    , 10^6]; (* bootstrap 1,000,000 times *)
ci[estimates_] := (Quantile[estimates, .95, interpolationType] -
     Quantile[estimates, .05, interpolationType]) / referenceIntervalWidth;
ciCorrect[estimates_] := Module[{n},
    n = Length@estimates;
    {#[[1]], #[[2]] / n} & /@ Tally[If[#[[3]] < percentile2p5, "actual is bigger",
         If[#[[1]] > percentile2p5, "actual is lower", "correct"]] & /@ estimates]
  ];
{
 <|"npts" → n, "ρ" → ρ, "percentWithTwoEyes" -> percentWithTwoEyes,
   "estimator" -> "2.5 percentile", "eyes" → "both",
   "90%CIPerRIWidth" → ci[t[[All, 1]]],
   "90%CI improvement factor over one eye" → ci[t[[All, 2]]] / ci[t[[All, 1]]]|>,

 <|"npts" → n, "ρ" → ρ, "percentWithTwoEyes" -> percentWithTwoEyes,
   "estimator" -> "2.5 percentile", "eyes" → "one",
   "90%CIPerRIWidth" → ci[t[[All, 2]]],
   "90%CI improvement factor over one eye" → ci[t[[All, 2]]] / ci[t[[All, 2]]]|>,

 <|"npts" → n, "ρ" → ρ, "percentWithTwoEyes" -> percentWithTwoEyes,
   "estimator" -> "2.5 percentile", "eyes" → "Duplicate missing",
   "90%CIPerRIWidth" → ci[t[[All, 3]]],
   "90%CI improvement factor over one eye" → ci[t[[All, 2]]] / ci[t[[All, 3]]]|>,

 <|"npts" → n, "ρ" → ρ,
   "percentWithTwoEyes" -> percentWithTwoEyes, "estimator" -> "mean", "eyes" → "both",
   "90%CIPerRIWidth" → ci[t[[All, 4]]],
   "90%CI improvement factor over one eye" → ci[t[[All, 2]]] / ci[t[[All, 1]]]|>,

 <|"npts" → n, "ρ" → ρ,
   "percentWithTwoEyes" -> percentWithTwoEyes, "estimator" -> "mean", "eyes" → "one",
   "90%CIPerRIWidth" → ci[t[[All, 5]]],
   "90%CI improvement factor over one eye" → ci[t[[All, 2]]] / ci[t[[All, 2]]]|>,

 <|"npts" → n, "ρ" → ρ, "percentWithTwoEyes" -> percentWithTwoEyes,
   "estimator" -> "mean", "eyes" → "Duplicate missing",
   "90%CIPerRIWidth" → ci[t[[All, 6]]],
   "90%CI improvement factor over one eye" → ci[t[[All, 2]]] / ci[t[[All, 3]]]|>
}
, {ρ , 0, 1, .05}
, {n, {40, 80, 120, 160}}
```

```
      , {percentWithTwoEyes, {.75, 1}}];]
sf2[num_] := NumberForm[num, 2];
```

Out[473]= {414.625, Null}

```
(* save data offline so that don't have to recompute *)
SetDirectory["X:\\RIM\\science\\reference intervals"];
Export["rmsTwoEyes3.m", rmsTwoEyes]
```

Out[476]= rmsTwoEyes3.m

In[477]:=
```
(*rmsTwoEyes=Get["X:\\RIM\\science\\reference intervals\\rmsTwoEyes3.m"];*)
```

In[478]:=
```
fig4 = Legended[Row[Table[
    dat = Select[rmsTwoEyes,
       #["estimator"] == "2.5 percentile" && #["percentWithTwoEyes"] == percentWithTwoEyes &&
         #["npts"] ≥ 40 && MemberQ[{"both", "Duplicate missing", "one"}, #["eyes"]] &][
      GroupBy[{#["eyes"], #["npts"]} &], All, {#["ρ"], 100. #["90%CIPerRIWidth"]} &];
dasher = Normal@Map[If[#[[1]] == "one", Dashing[{}], If[#[[1]] == "both", Dashed,
          If[#[[1]] == "Duplicate missing", Directive[Dotted, Thick], Dashed]]] &, Keys@dat];
colorer = Normal[ColorData[97][Floor[#[[2]] / 40 + 1]] & /@ Keys@dat];
styler = Table[Directive[dasher[[i]], colorer[[i]]], {i, Length@dasher}];
ListPlot[Values@dat,
 PlotStyle -> styler, Frame → True, FrameLabel → {"Correlation between eyes (r)",
       If[percentWithTwoEyes != 1, "90% CI / RI\nfor 2.5th percentile", None]}
     , PlotLabel → StringForm["``% with 2 eyes", Round[100 percentWithTwoEyes]],
     ImageSize → {Automatic, 250}
, Joined -> True, LabelStyle → Directive[Black, 12], PlotRange → {10, 36}, PlotRangePadding → None,
     FrameTicks → {{Join[{#, If[percentWithTwoEyes == 1, "", StringForm["``%", #]], {.015, 0}} & /@
           {10, 15, 20, 30, 40, 50}, {#, "", {0.005, 0}} & /@ Range[100]],
         Join[{#, "", {.015, 0}} & /@ {10, 15, 20, 30, 40, 50}, {#, "", {0.005, 0}} & /@ Range[100]]},
       {Join[{#, #, {.015, 0}} & /@ {If[percentWithTwoEyes ≠ 1, 0, 0], .2, .4, .6, .8, 1},
          {#, "", {.005, 0}} & /@ Range[0, 1, .05]]
         , Join[{#, "", {.015, 0}} & /@ {0, .2, .4, .6, .8, 1}, {#, "", {.005, 0}} & /@ Range[0, 1, .05]]}
      }, PlotLabels →
      If[percentWithTwoEyes == 1, Normal /@ {StringForm["n=``", Normal@Keys[dat][[1, 2]]],
         None, None, None, None, Keys[dat][[4, 2]], None, None, Keys[dat][[7, 2]],
         None, None, Keys[dat][[10, 2]], None, None, Keys[dat][[13, 2]]}
       , None]
     , PlotRange -> All]
   , {percentWithTwoEyes, {.75, 1}
    (*DeleteDuplicates@Normal@rmsTwoEyes[All,"percentWithTwoEyes"]*)}]
  , "    "
 ]
 , Placed[
  LineLegend[{Dashing[{}], Dashed, Dotted}, {"One eye", "Use all available", "Duplicate missing"},
   LegendLayout → "Row", LegendMarkerSize → 30, LegendMargins → {{0, 0}, {0, 1}}
  ], Below]
]
```

Out[478]=

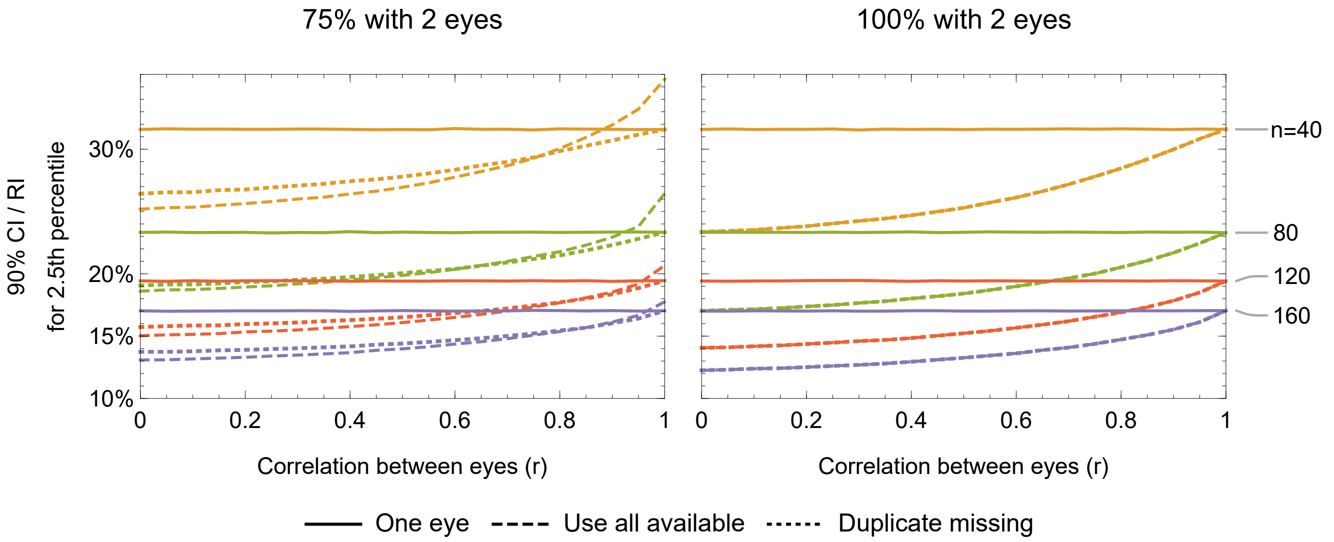

In[479]:=
```
Export[StringTemplate["X:\\RIM\\science\\reference intervals\\figures\\Fig 4.``"][#],
    fig4] & /@ {"pdf", "eps"};
```

The following figure shows that when estimating the mean (for example, for the reliability coefficient), using all available eyes without duplicating missing eyes has effectively equivalent performance. Without any knowledge of if variability between eyes is correlated, it seems simplest just to use all available eyes, as recommended in the paper.

In[480]:=
```
fig4a = Legended[Row[Table[
    dat = Select[rmsTwoEyes, #["estimator"] == "mean" && #["percentWithTwoEyes"] == percentWithTwoEyes &
         #["npts"] ≥ 40 && MemberQ[{"both", "Duplicate missing", "one"}, #["eyes"]] &][
       GroupBy[{#["eyes"], #["npts"]} &], All, {#["ρ"], 100. #["90%CIPerRIWidth"]} &];
dasher = Normal@Map[If[#[[1]] == "one", Dashing[{}], If[#[[1]] == "both", Dashed,
          If[#[[1]] == "Duplicate missing", Directive[Dotted, Thick], Dashed]]] &, Keys@dat];
colorer = Normal[ColorData[97][Floor[#[[2]] / 40 + 1]] & /@ Keys@dat];
styler = Table[Directive[dasher[[i]], colorer[[i]]], {i, Length@dasher}];
ListPlot[Values@dat,
 PlotStyle -> styler, Frame → True, FrameLabel →
      {"Correlation between eyes (r)", If[percentWithTwoEyes != 1, "90% CI / RI\nfor mean", None]}
     , PlotLabel → StringForm["``% with 2 eyes", Round[100 percentWithTwoEyes]],
     ImageSize → {Automatic, 250}
, Joined -> True, LabelStyle → Directive[Black, 12], PlotRange → {0, 16}, PlotRangePadding → None,
     FrameTicks → {{Join[{#, If[percentWithTwoEyes == 1, "", StringForm["``%", #]], {.015, 0}} & /@
          {5, 10, 15, 20, 30, 40, 50}, {#, "", {0.005, 0}} & /@ Range[100]], Join[
        {#, "", {.015, 0}} & /@ {5, 10, 15, 20, 30, 40, 50}, {#, "", {0.005, 0}} & /@ Range[100]]},
      {Join[{#, #, {.015, 0}} & /@ {If[percentWithTwoEyes ≠ 1, 0, 0], .2, .4, .6, .8, 1},
        {#, "", {.005, 0}} & /@ Range[0, 1, .05]]
       , Join[{#, "", {.015, 0}} & /@ {0, .2, .4, .6, .8, 1}, {#, "", {.005, 0}} & /@ Range[0, 1, .05]]}
      }, PlotLabels →
      If[percentWithTwoEyes == 1, Normal /@ {StringForm["n=``", Normal@Keys[dat][[1, 2]]],
         None, None, None, None, Keys[dat][[4, 2]], None, None, Keys[dat][[7, 2]],
         None, None, Keys[dat][[10, 2]], None, None, Keys[dat][[13, 2]]}
       , None]
     , PlotRange -> All]
    , {percentWithTwoEyes, {.75, 1}
     (*DeleteDuplicates@Normal@rmsTwoEyes[All,"percentWithTwoEyes"]*)}]
   , "    "
  ]
  , Placed[
   LineLegend[{Dashing[{}], Dashed, Dotted}, {"One eye", "Use all available", "Duplicate missing"},
    LegendLayout → "Row", LegendMarkerSize → 30, LegendMargins → {{0, 0}, {0, 1}}
   ], Below]
 ]
```

Out[480]=

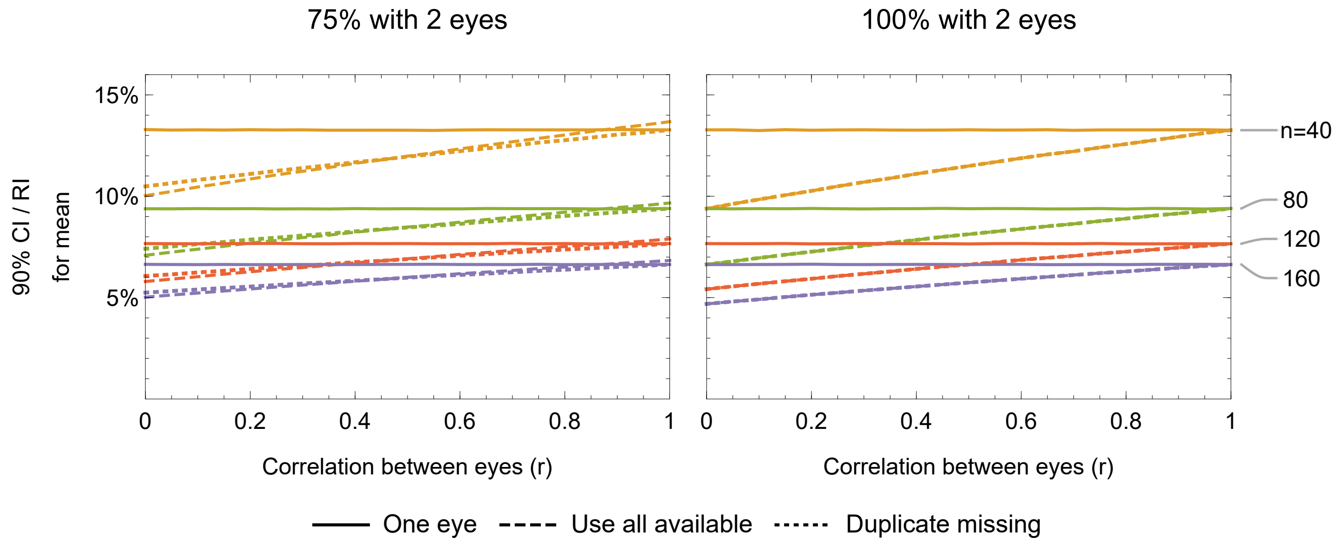

Other analysis

How many “inconclusive” results are there at CI = 20% RI?

Assuming a Gaussian distribution, what fraction of normal subjects would be inconclusive based on estimated errors in the RI (assuming there isn’t actually any error).

In[481]:=
```
fractionInconclusive[ciFraction_] := Module[{RIWIdth = 2 × 1.96},
  2 (CDF[NormalDistribution[], -1.96 + ciFraction RIWIdth] -
     CDF[NormalDistribution[], -1.96 - ciFraction RIWIdth])]
```

In[482]:=
```
Row[
 Table[
  With[
   {RIWIdth = 2 × 1.96},
   Plot[100 fractionInconclusive[x / 100],
    {x, 0, 35}, AxesLabel → {"CI/RI %", "% normal subjects\ninconclusive"},
    LabelStyle → Directive[Black, 12], ImageSize → 350, PlotRange → {0, 60},
    Epilog → {Black, Thin,
      Line[{{100 ciFraction, 0}, {100 ciFraction, 100 fractionInconclusive[ciFraction]},
        {0, 100 fractionInconclusive[ciFraction]}}]
      , Inset[
       Show[{Plot[PDF[NormalDistribution[], x], {x, -3.5, 3.5}, PlotLabel → StringForm["``%",
            Round[100 fractionInconclusive[ciFraction]]], ImageSize → 100, PlotRange → {0, .4},
          Epilog → {Black, Line[{{-1.96, 0}, {-1.96, PDF[NormalDistribution[], -1.96]}}],
            Line[{{+1.96, 0}, {+1.96, PDF[NormalDistribution[], +1.96]}}]}
          , Axes → False
         ],

         Plot[PDF[NormalDistribution[], x], {x, -1.96 - ciFraction RIWIdth,
           -1.96 + ciFraction RIWIdth}, Filling → Bottom, PlotRange → {0, .4}],
         Plot[PDF[NormalDistribution[], x], {x, +1.96 - ciFraction RIWIdth,
           +1.96 + ciFraction RIWIdth}, Filling → Bottom, PlotRange → {0, .4}]
        }
       ]
       ,
       {50 ciFraction, 100 fractionInconclusive[ciFraction]}, {Center, Bottom}]
     }]
  ],
  {ciFraction, {.1, .2, .25}}]
]
```

Out[482]=

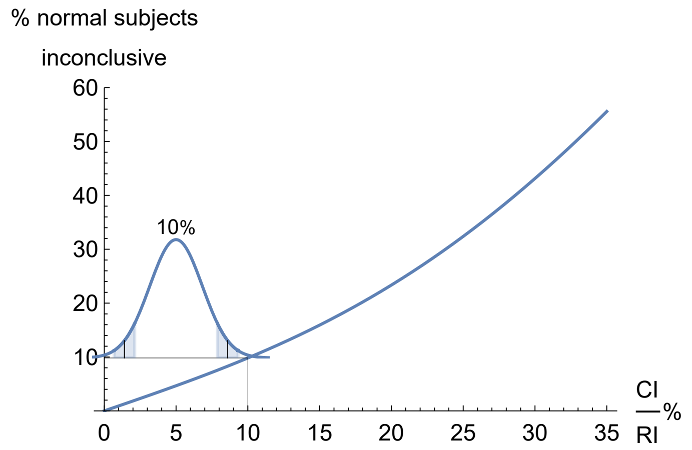

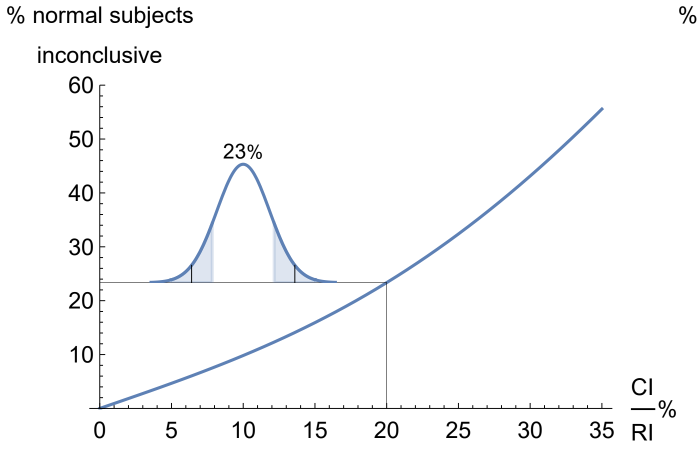

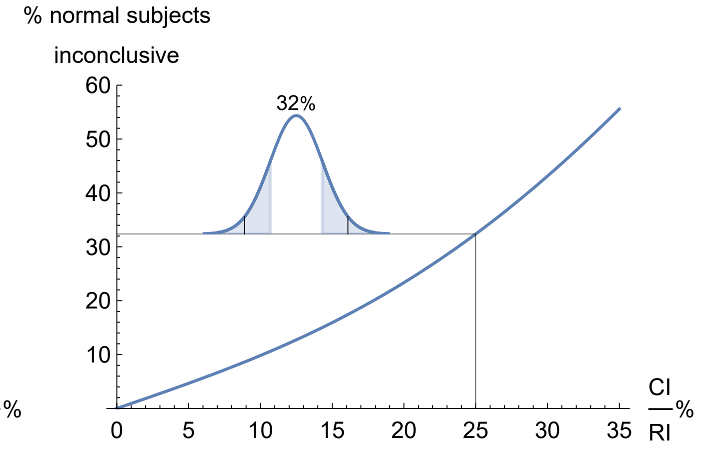

bootstrap individuals vs. eyes.

Plotted below is the percent of times that the 90% CI of the reference limit actually contains the true reference limit in the cases of bootstrapping eyes (solid lines) or individuals (dashed lines). Bootstrapping individuals is better, being closer to 90% for all cases.

In[484]:=

```
(* bootstrap pairs, returns 5th, 50th, and 95th percentiles for the desired quantile *)
bootMethod2[data_, quant_] := Module[{boot},
   boot = ParallelTable[
     Quantile[Flatten@RandomChoice[data, Length@data], quant, interpolationType], 10^3];
   {Quantile[boot, .05, interpolationType], Quantile[boot, .5, interpolationType],
    Quantile[boot, .95, interpolationType]}
  ];
μ1 = 1 ;
μ2 = 1;
σ = 2;
Timing[eyesOrPeople = Dataset@Flatten@Table[
      trueMean = μ1;
      trueSD = σ;
      percentile2p5 = InverseCDF[NormalDistribution[trueMean, trueSD], .025];
      referenceIntervalWidth =
       InverseCDF[NormalDistribution[trueMean, trueSD], .975] - percentile2p5;
      x1 = NormalDistribution[0, 1];
```

```
x2 = NormalDistribution[0, 1];
t = Table[
  rvx1 = RandomVariate[x1, n];
  rvx2 = RandomVariate[x2, n];
  y1 = μ1 + σ rvx1;
  y2 = μ2 + σ (ρ rvx1 + Sqrt[(1 - ρ^2)] rvx2);
  data = Join[Transpose[{y1[[1 ;; Floor[percentWithTwoEyes n]]],
       y2[[1 ;; Floor[percentWithTwoEyes n]]]}], y1[[Floor[percentWithTwoEyes n] + 1 ;; -1]]];
  duplicator = If[Length@# > 0, #, {#, #}] & /@ data;
  (*meaner = Mean@Flatten@#&/@data;*)
  {Quantile[Flatten@data, .025, interpolationType] - percentile2p5
   , (*2*)Quantile[y1, .025, interpolationType] - percentile2p5
   (*,Quantile[meaner,.025,interpolationType]-percentile2p5*)
   , (*3*)Quantile[Flatten@duplicator, .025, interpolationType] - percentile2p5
   , (*4*)bootMethod[Flatten@data, .025]
   , (*5*)bootMethod[y1, .025](* ,bootMethod[meaner,.025]*)
   , (*6*)bootMethod[Flatten@duplicator, .025]
   , (*7*)bootMethod2[data, .025]
   , (*8*)bootMethod2[duplicator, .025]
  }
  , 10^3];
ci[estimates_] := (Quantile[estimates, .95, interpolationType] -
    Quantile[estimates, .05, interpolationType]) / referenceIntervalWidth;
ciCorrect[estimates_] := Module[{n},
  n = Length@estimates;
  {#[[1]], #[[2]] / n} & /@ Tally[If[#[[3]] < percentile2p5, "actual is bigger",
       If[#[[1]] > percentile2p5, "actual is lower", "correct"]] & /@ estimates]
 ];
{
 <|"npts" → n, "ρ" → ρ, "percentWithTwoEyes" -> percentWithTwoEyes,
  "estimator" -> "2.5 percentile", "eyes" → "All available",
  "90%CIPerRIWidth" → ci[t[[All, 1]]],
  "90%CI improvement factor over one eye" → ci[t[[All, 2]]] / ci[t[[All, 1]]],
  "90%CI compared to actual, boot eyes" → ciCorrect[t[[All, 4]]],
  "90%CI compared to actual, boot individuals" → ciCorrect[t[[All, 7]]]|>,

 <|"npts" → n, "ρ" → ρ, "percentWithTwoEyes" -> percentWithTwoEyes,
  "estimator" -> "2.5 percentile", "eyes" → "One eye only",
  "90%CIPerRIWidth" → ci[t[[All, 2]]],
  "90%CI improvement factor over one eye" → ci[t[[All, 2]]] / ci[t[[All, 2]]],
  "90%CI compared to actual, boot eyes" → ciCorrect[t[[All, 5]]],
  "90%CI compared to actual, boot individuals" → ciCorrect[t[[All, 5]]]|>,

 <|"npts" → n, "ρ" → ρ, "percentWithTwoEyes" -> percentWithTwoEyes,
  "estimator" -> "2.5 percentile", "eyes" → "Duplicate missing",
  "90%CIPerRIWidth" → ci[t[[All, 3]]],
  "90%CI improvement factor over one eye" → ci[t[[All, 2]]] / ci[t[[All, 3]]],
  "90%CI compared to actual, boot eyes" → ciCorrect[t[[All, 6]]],
  "90%CI compared to actual, boot individuals" → ciCorrect[t[[All, 8]]]|>
}
, {ρ , 0, 1, .25}
, {n, {40, 80, 120, 160}}
, {percentWithTwoEyes, {.75, 1}}];]
sf2[num_] := NumberForm[num, 2];
```

Out[488]= {5547.67, Null}

In[490]:=
```
getType[list_, type_] := Module[{}, Select[list, StringMatchQ[#[[1]], type] &][[1, 2]]];
Column@Table[Column[{
    Style[type, 20],
    Row@Table[
      dat = Select[eyesOrPeople, #["estimator"] == "2.5 percentile" && #["percentWithTwoEyes"] ==
          .75 && #["npts"] ≥ 40 && MemberQ[{(*"both",*)type(*,"one"*)}, #["eyes"]] &];
      dat = Join[dat[All, <|"ρ" -> #["ρ"], "npts" → #["npts"],
            "boot method" → "eyes", "90%CI compared to actual" ->
             100 (getType[#["90%CI compared to actual, boot eyes"], "correct"])|> &]
          , dat[All, <|"ρ" -> #["ρ"], "npts" → #["npts"], "boot method" → "individuals",
            "90%CI compared to actual" ->
             100 (getType[#["90%CI compared to actual, boot individuals"], "correct"])|> &]
         ][GroupBy[{#["boot method"], #["npts"]} &], All,
         {#["ρ"], #["90%CI compared to actual"]} &];
dasher = Normal@Map[If[#[[1]] == "eyes", Dashing[{}], If[#[[1]] == "individuals",
            Dashed, If[#[[1]] == "Duplicate missing", Dotted, Dashed]]] &, Keys@dat];
colorer = Normal[ColorData[97][Floor[#[[2]] / 40 + 1]] & /@ Keys@dat];
styler = Table[Directive[dasher[[i]], colorer[[i]]], {i, Length@dasher}];
ListPlot[Values@dat,
 PlotStyle -> styler, Frame → True, FrameLabel → {"Correlation between eyes (r)",
          If[percentWithTwoEyes != 1, "% time CI is correct\nfor 2.5th percentile", None]}
        , PlotLabel → StringForm["``% with 2 eyes", Round[100 percentWithTwoEyes]],
        ImageSize → {Automatic, 200}
, Joined -> True, LabelStyle → Directive[Black, 12], PlotRange → {50, 95}, PlotRangePadding → None,
        FrameTicks → {{Join[{#, If[percentWithTwoEyes == 1, "", StringForm["``%", #]], {.015, 0}} & /@
             {50, 60, 70, 80, 90, 100}, {#, "", {0.005, 0}} & /@ Range[100]], Join[
            {#, "", {.015, 0}} & /@ {50, 60, 70, 80, 90, 100}, {#, "", {0.005, 0}} & /@ Range[100]]},
          {Join[{#, #, {.015, 0}} & /@ {If[percentWithTwoEyes ≠ 1, 0, .2], .2, .4, .6, .8, 1},
            {#, "", {.005, 0}} & /@ Range[0, 1, .05]]
           , Join[{#, "", {.015, 0}} & /@ {0, .2, .4, .6, .8, 1},
            {#, "", {.005, 0}} & /@ Range[0, 1, .05]]}

          }, PlotLabels → If[percentWithTwoEyes == 1,
          Normal /@ {StringForm["n=``", Normal@Keys[dat][[1, 2]]], Normal@Keys[dat][[2, 2]],
             Normal@Keys[dat][[3, 2]], Normal@Keys[dat][[4, 2]], None, None, None, None}
          , None],
        GridLines → {None, {90}}, PlotLegends → If[percentWithTwoEyes == 1,
          LineLegend[{Dashing[{}], Dashed, Dotted}, {"eyes", "individuals"}
           , LegendLabel → "Bootstrap", LegendFunction -> Framed], None]
        , PlotRange -> All]
      , {percentWithTwoEyes, {.75, 1}
        (*DeleteDuplicates@Normal@rmsTwoEyes[All,"percentWithTwoEyes"]*)}]
    }, Center],
  {type, DeleteDuplicates@Normal@eyesOrPeople[All, "eyes"]}]
```

Out[491]=

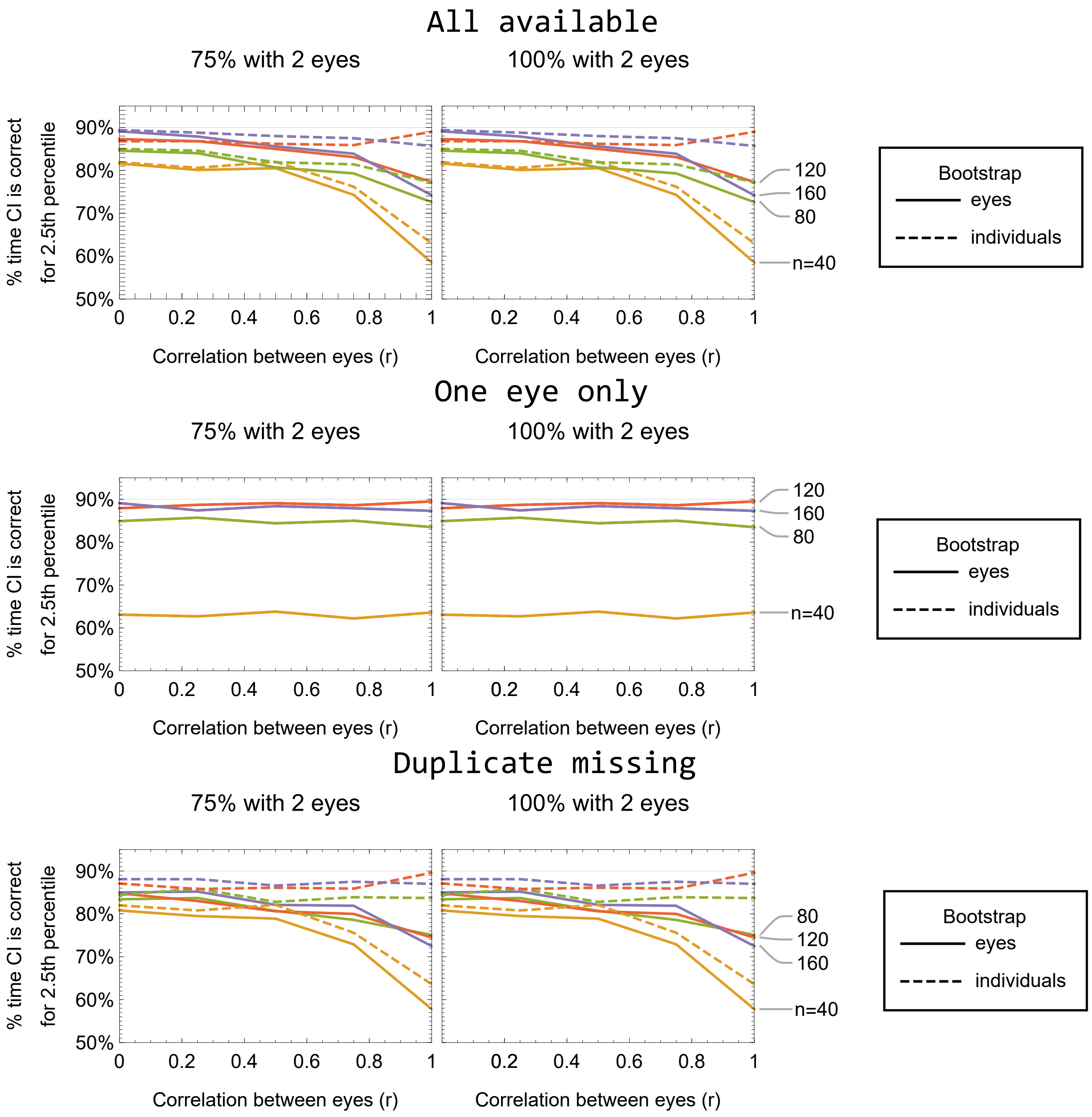

Tukey' s fences

Far outliers are 3 IQR, rejecting this many subjects in a Gaussian distribution

In[492]:=
```
2 (1 - CDF[NormalDistribution[],
      Quartiles[NormalDistribution[]][[3]] + 3 InterquartileRange[NormalDistribution[]]] // N)
```

Out[492]= 2.34194×10^{-6}

In the Gamma distribution used in Fig 1 and 3, far outliers will reject

In[493]:=
```
1 - CDF[sampleDist, Quartiles[sampleDist][[3]] + 3 InterquartileRange[sampleDist]] +
   CDF[sampleDist, Quartiles[sampleDist][[1]] - 3 InterquartileRange[sampleDist]] // N
```

Out[493]= 0.00183506
